# Phase transfer studies of iron oxide nanoparticles: characterization based insights for applications

**DOI:** 10.1039/d6na00313c

**Published:** 2026-08-19

**Authors:** Haroon Zafar, Vyshnav Punnath Sivasankaran, Tina Bergh, Sawssen Slimani, Sjoerd Hak, Davide Peddis, Sulalit Bandyopadhyay

**Affiliations:** a Particle Engineering Centre, Department of Chemical Engineering, Norwegian University of Science and Technology (NTNU) Trondheim Norway sulalit.bandyopadhyay@ntnu.no; b Department of Chemical Engineering, Norwegian University of Science and Technology (NTNU) Trondheim Norway; c Institute of Structure of Matter, National Research Council, nM2-lab Via Salaria km 29.300, Monterotondo Scalo 00015 Roma Italy; d Department of Chemistry, Industrial Chemistry & Genova INSTM RU, nM2-Lab, University of Genova 16146 Genova Italy; e Department of Biotechnology and Nanomedicine, SINTEF Industry Norway; f Department of Circulation and Medical Imaging, Norwegian University of Science and Technology (NTNU) Trondheim Norway

## Abstract

This study systematically explores the synthesis and functionalization of iron oxide nanoparticles (IONPs) synthesized *via* thermal decomposition methods using three common precursors: iron(iii) oleate (FeOl), iron pentacarbonyl [Fe(CO)_5_], and iron acetylacetonate [Fe(acac)_3_]. Comprehensive characterization was employed to evaluate the morphology, crystal structure, and magnetic properties of the synthesized IONPs, alongside four phase transfer strategies. Among the investigated methods, Fe(acac)_3_-based synthesis predominantly yielded Fe_3_O_4_ nanoparticles with superior crystallinity and magnetic properties (112 A m^2^ per kg of Fe). However, the synthesis was limited by solvent instability during thermal decomposition. FeOl-based synthesis offered better control over particle size and morphology but resulted in mixed-phase nanoparticles (Fe_3_O_4_ and γ-Fe_2_O_3_) with suboptimal magnetization. In contrast, Fe(CO)_5_ produced nanoparticles with significantly reduced magnetic performance. While none of the four evaluated phase transfer methods (oxidative cleavage, base bath, ligand exchange using sodium citrate, and tetramethylammonium hydroxide) significantly altered the magnetization of the IONPs, oxidative cleavage and base bath methods were preferred. Their operational simplicity, reproducibility, and suitability for processing larger batches (up to 200 mg IONPs) make them particularly attractive from a scalability perspective compared to ligand-exchange-based methods. Selected phase-transferred nanoparticles demonstrate potential applications as MRI contrast agents. By systematically comparing synthesis approaches and phase transfer techniques, this work identifies critical factors that influence IONP properties and provides valuable insights for designing nanoparticles optimized for biomedical applications.

## Abstract 2

This study systematically explores the synthesis and functionalization of iron oxide nanoparticles (IONPs) synthesized *via* thermal decomposition methods using three common precursors: iron(iii) oleate, iron pentacarbonyl, and iron acetylacetonate.

## Graphical abstract

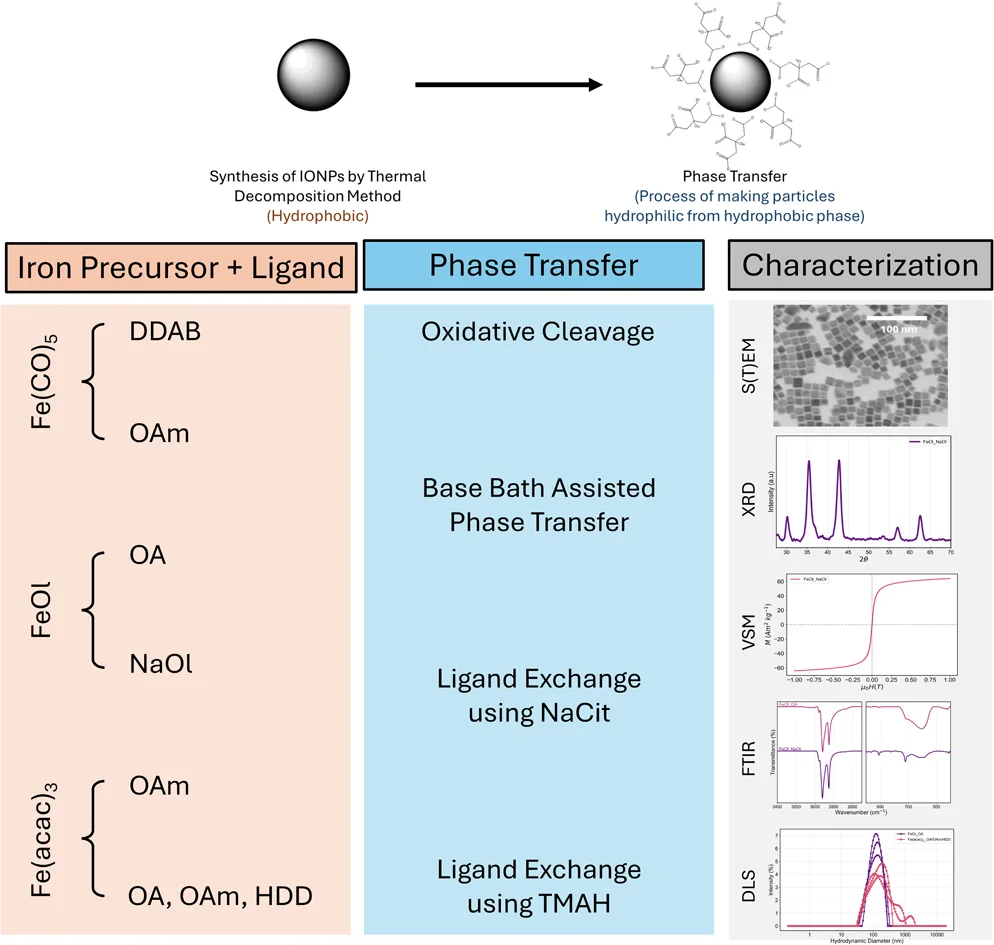

## 1 Introduction

In recent years, iron oxide nanoparticles (IONPs) have captured significant interest in the field of biomedicine due to their superparamagnetic properties, high biocompatibility, and ease of surface functionalization. The versatility of IONPs has made them essential in a wide array of applications, ranging from cutting-edge medical treatment to environmental remediation and beyond.^1^ The recent increase in interest around IONPs has led to the rapid development of various synthesis methods, resulting in IONPs with diverse physicochemical characteristics.

Among these methods, co-precipitation is widely recognized for its cost-effectiveness and scalability. However, achieving precise control over key physicochemical properties, such as size, morphology, and magnetic behavior, remains a significant challenge with this approach.^2^ In contrast, thermal decomposition has emerged as a preferred synthesis method, offering precise control over particle attributes. This technique consistently produces nanoparticles with narrow size distributions, well-defined shapes, high magnetization, and superior crystallinity.^3^ However, these advantages come with certain challenges, especially for biomedical applications. In the thermal decomposition method, IONPs are often coated with organic molecules such as oleic acid (OA) or oleylamine (OAm), which render the particles hydrophobic. Although hydrophobic nanoparticles can be suitable for certain applications, their use in biomedicine generally demands colloidal stability in aqueous environments. For example, citrate-coated hydrophilic IONPs are widely used as MRI contrast agents to improve tumor imaging.^4^ In hyperthermia therapy, hydrophilic particles ensure efficient heat generation in cancerous tissues when exposed to alternating magnetic fields.^5^ In addition, applications such as biosensing and gene therapy rely on surface properties that allow efficient interaction with biological systems.^6^ Therefore, modifying the surface of IONPs from hydrophobic to hydrophilic becomes important for ensuring high application potential in biomedical fields.

Several techniques have been employed to enhance the water dispersibility of IONPs, each offering its unique set of advantages and drawbacks. Among these, ligand exchange is a method in which the surface ligands of IONPs are partially or completely replaced with compounds that render the IONPs hydrophilic.^7^ However, this approach demands an excess of ligand molecules and involves complex reaction conditions. Alternatively, bilayers composed of amphiphilic molecules can be formed to counteract the hydrophobic tendencies of organic ligands.^8^ Nevertheless, it is essential to note that this amphiphilic polymer coating may increase the hydrodynamic size (*H*_d_) of the nanoparticles, which could limit their suitability for applications requiring direct water interaction.^9^ An additional strategy for transferring IONPs involves the oxidative cleavage of unsaturated ligands covering the nanoparticle surface. However, one drawback of these oxidation routes is that they cannot oxidize the surface ligand completely, and this frequently leads to the aggregation of nanoparticles.^10,11^

Our present study systematically investigates the synthesis and phase-transfer of IONPs, evaluating six thermal decomposition methods and four phase-transfer strategies. Using three widely employed iron precursors, Fe(CO)_5_, FeOl, and Fe(acac)_3_, we assess their impact on nanoparticle formation, monodispersity, and stability. Comprehensive characterization using electron microscopy, light scattering, and spectroscopic techniques provides detailed insight into their physico-chemical properties before and after phase-transfer. A central motivation for this work is the often-overlooked interdependence between nanoparticle synthesis and phase-transfer. Although thermal decomposition methods reliably produce highly uniform, ultrasmall, and strongly magnetic IONPs, subsequent phase-transfer steps may substantially modify these desirable properties.^12^ For instance, encapsulation approaches based on amphiphilic polymers or silica shells often increase the *H*_d_ size and may attenuate the magnetic response, including performance in MRI applications. Conversely, ligand-exchange and chemical reaction based strategies can better preserve intrinsic material properties but often suffer from low phase transfer efficiency, limited scalability, and poor reproducibility.^9^ By systematically exploring combinations of synthesis and phase-transfer methods, this study provides a practical framework to guide the rational selection and optimization of IONPs tailored to specific biomedical requirements. To show the potential of these IONPs, we have further investigated the performance of selected IONPs for MRI applications.

## 2 Materials and methods

### 2.1 Materials

Iron(iii) chloride hexahydrate (FeCl_3_·6H_2_O), iron pentacarbonyl [Fe(CO)_5_], 1-octadecene (ODE, technical grade, 90%), didecyldimethylammonium bromide (DDAB, 98%), iron acetylacetonate [Fe(acac)_3_], benzyl ether (98%), oleic acid (OA, technical grade, 90%), 1,2-hexadecanediol (HDD, technical grade, 90%), oleylamine (OAm, technical grade, 70%) were obtained from Merck Life Science/Sigma-Aldrich Norway AS. Sodium oleate (NaOl, 97%), ethanol (96 vol%), *n*-hexane (≥95%), acetone (≥99%), and isopropyl alcohol (≥98%) were purchased through VWR. Polyvinylpyrrolidone (PVP, molecular weight 40 000 g mol^−1^), potassium permanganate (KMnO_4_, ACS grade), tetramethylammonium hydroxide (TMAH, 25 wt% in water), sodium periodate (NaIO_4_, 99.8%), potassium hydroxide (KOH, reagent grade), and potassium carbonate (K_2_CO_3_, reagent grade) were all procured from Sigma-Aldrich. Milli-Q water (18.2 MΩ cm resistivity) was used for all experiments.

### 2.2 Method: synthesis of IONPs

Unless otherwise specified, all thermal decomposition reactions were carried out in a 100 mL three-neck round-bottom flask placed in a heating mantle and connected to a Schlenk line to maintain an inert atmosphere. The flask was fitted with a magnetic stir bar, a condenser, and a thermometer adapter holding a thermocouple. The thermocouple was connected to a temperature controller to ensure precise thermal regulation throughout the reaction.

#### 2.2.1 Thermal decomposition of Fe(CO)_5_ + DDAB

The following method was adapted from the protocol described by Singh *et al.*^13^ In a three-neck round-bottom flask, 25 mL of ODE and 325 mg of DDAB (0.8 mmol) were added. The mixture was magnetically stirred at 700 rpm and degassed at 120 °C under an argon atmosphere for 30 minutes. After degassing, the temperature was increased to 180 °C, and 1.4 mL (10.36 mmol) of Fe(CO)_5_, was injected through the septa to maintain an inert atmosphere and prevent air exposure of the precursor. The reaction was held at this temperature for 30 minutes before the temperature was further raised to 220 °C at a rate of 10 °C min^−1^ and maintained for 2 hours. The solution was cooled to room temperature upon completion, and the magnetic stir bar was washed with hexane. Acetone was added to precipitate the nanoparticles, which were magnetically separated, and the supernatant was discarded. The nanoparticles were washed twice by redispersing them in hexane and re-precipitating with acetone. The nanoparticles were dispersed in toluene for long-term storage.

#### 2.2.2 Thermal decomposition of Fe(CO)_5_ + OAm

The following method was adapted from the protocol described by Peng *et al.*^14^ A solution consisting of 50 mL of ODE and 740 µL (2.34 mmol) of OAm was prepared and degassed under an argon atmosphere at 120 °C for 30 minutes with continuous magnetic stirring. Following degassing, the temperature was increased to 180 °C, and 1.8 mL (13.32 mmol) of Fe(CO)_5_ was injected into the hot solution. The reaction mixture was maintained at 180 °C for 20 minutes before being cooled to room temperature. Once cooled down, the supernatant was discarded, and nanoparticles were washed off the magnet using 20 mL hexane. The IONPs were purified and stored by following the procedure described above in Section 2.2.1.

#### 2.2.3 Thermal decomposition of Fe(acac)_3_ + OAm

This method was adopted with some modifications from the work of Xu *et al.*^15^ In a three-neck round bottom flask, 1.06 g (3 mmol) of Fe(acac)_3_ was dissolved in a mixture of 20 mL (60.6 mmol) of OAm and 10 mL of benzyl ether. The reaction mixture was degassed at 110 °C for 1 hour under a nitrogen atmosphere. Following degassing, the temperature was increased to 300 °C at a heating rate of 10 °C min^−1^. The reaction was refluxed at this temperature for 1 hour before being cooled to room temperature. The resulting IONPs were precipitated, washed three times with 50 mL of ethanol, and magnetically separated after each washing step. For long-term storage, the IONPs were dispersed in toluene.

#### 2.2.4 Thermal decomposition of Fe(acac)_3_ + OA/OAm/HDD

This synthesis was adopted with some modifications from Yang *et al.*^16^ In a three-neck round-bottom flask, 0.353 g (1 mmol) of Fe(acac)_3_ and 2.1 g (8 mmol) of HDD were dissolved in a mixture of 5.08 mL (16 mmol) of OA, 1.32 mL (4 mmol) of OAm, and 20 mL of benzyl ether. The reaction mixture was degassed under an argon atmosphere at 110 °C for 1 hour. After degassing, the temperature was increased to 200 °C and held for 30 minutes. The mixture was then heated to reflux at 290 °C with a controlled heating rate of 10 °C min^−1^ and maintained at this temperature for 1 hour. The IONPs were purified and stored by following the procedure described above in Section 2.2.3.

#### 2.2.5 Thermal decomposition of FeOleate + OA

Iron oleate was synthesized prior to nanoparticle preparation, following a literature protocol with minor modifications.^3^ In a 250 mL round-bottom flask, 25 mL of Milli-Q water, 70 mL of hexane, and 40 mL of ethanol were combined and stirred at 600 rpm. FeCl_3_·6H_2_O (5.395 g) and NaOl (18.25 g) were added sequentially, and the mixture was refluxed at 70 °C for 4 h. After cooling to room temperature, the organic phase was separated, washed three times with Milli-Q water, and concentrated under reduced pressure using rotary evaporator to yield FeOl as a dark-brown viscous liquid which was stored at 4 °C until use.

FeOleate + OA synthesis was adapted from the work of Sharma *et al.*^17^ In a three-neck round-bottom flask, 1.6 g of FeOl and 600 µL (1.9 mmol) of OA were dissolved in 25 mL of ODE under an argon atmosphere. The solution was then heated to reflux at 318 °C with a controlled heating rate of 3 °C min^−1^. The reaction was maintained at reflux for 45 minutes, before the solution was cooled down to room temperature. The IONPs were precipitated with a mixture of acetone and isopropyl alcohol (2 : 1, v/v) and magnetically separated. The supernatant was discarded, and the IONPs were further washed thrice with acetone and dispersed in toluene for long-term storage.

#### 2.2.6 Thermal decomposition of Fe oleate + Na oleate

This synthesis was also adopted from the work of Sharma *et al.*^17^ In a three-neck round-bottom flask, 0.83 g of FeOl and 213 mg of NaOl were dissolved in 14 mL of ODE under an argon atmosphere. The solution was then heated to reflux at 318 °C with a controlled heating rate of 2.8 °C min^−1^. The reaction was maintained at reflux for 45 minutes before the solution was cooled to room temperature. The IONPs were purified and stored by following the procedure described above in Section 2.2.5.

In this manuscript, synthesized IONPs are labeled according to the combination of iron precursor and surface ligand employed during synthesis, as summarized in Table 1. This labeling scheme is adopted throughout the manuscript.

**Table 1:** Sample labeling scheme for IONPs synthesized by thermal decomposition indicating the iron precursor and surface ligand(s) used in each synthesis

Label	Iron precursor	Surface ligand(s)
FeOl_OA	Iron(iii) oleate	Oleic acid
FeOl_NaOl	Iron(iii) oleate	Sodium oleate
Fe(acac)_3__OAm	Iron acetylacetonate	Oleylamine
Fe(acac)_3__OA/OAm/HDD	Iron acetylacetonate	Oleic acid, oleylamine, 1,2-hexadecanediol
Fe(CO)_5__DDAB	Iron pentacarbonyl	Didecyldimethylammonium bromide
Fe(CO)_5__OAm	Iron pentacarbonyl	Oleylamine

### 2.3 Method: phase transferring hydrophobic IONPs to hydrophilic phase

Phase transfers used fixed input masses (50 mg for oxidative cleavage, base bath, and TMAH; 5 mg for NaCit); corresponding nominal Fe-based concentrations are given in the SI.

#### 2.3.1 Phase-transfer by oxidative reaction

IONPs stored in toluene were first precipitated by adding 50 mL of acetone and isopropyl alcohol mixture (2 : 1, v/v) and collected *via* magnetic decantation. The supernatant was discarded, and the precipitated nanoparticles were left in an open container inside a fume hood for several minutes to allow solvent evaporation. Subsequently, 50 mg of the dried IONPs were redispersed in 25 mL of cyclohexane. To this dispersion, 17.5 mL of tertiary butanol, 1.25 mL of a 5 wt% K_2_CO_3_ solution, 2.5 mL of a 40 wt% PVP solution, and 10 mL of an oxidizing agent solution (containing 4.5 mg of KMnO_4_ and 225 mg of NaIO_4_) were added. The mixture was magnetically stirred at 700 rpm for 2 hours. After the reaction, IONPs were collected magnetically, washed once with ethanol and twice with Milli-Q water by doing centrifugation at 10 000 rpm. All phase-transferred IONPs were finally dispersed in 10 mL Milli-Q water and stored in a refrigerator.^9^

#### 2.3.2 Base bath assisted phase-transfer

In a centrifuge tube, 50 mg of IONPs dispersed in 5 mL of toluene (10 mg mL^−1^) were mixed with an alkaline solution consisting of 20 mL isopropyl alcohol, 20 mL Milli-Q water, and 2 g KOH. The centrifuge tube was placed in an ultrasonication bath and sonicated for 30 minutes, with vortexing for 15 seconds every 5 minutes. After sonication, the mixture was shaken at 750 rpm for 24 hours. The phase-transferred IONPs were then magnetically separated by washing thrice with ethanol and twice with Milli-Q water. Finally, the phase-transferred particles were dispersed in 10 mL Milli-Q water and stored in a refrigerator.^18^

#### 2.3.3 Phase-transfer using Na citrate

IONPs were first washed by suspending 5 mg of IONPs in a mixture of 20 mL methanol and 5 mL acetone, followed by magnetic separation for 2 minutes. This process was repeated three times with acetone to remove any impurities. The IONPs were then dried by air flushing and subsequently dispersed in 10 mL of a 10 mM sodium citrate solution. The IONPs were sonicated using a probe sonicator for 20 minutes at 70% amplitude, followed by magnetic separation, and washed thrice with Milli-Q water. Finally, the phase-transferred particles were dispersed in 2 mL Milli-Q water and stored in a refrigerator.^17^

#### 2.3.4 Phase-transfer using TMAH

50 mg of IONPs were transferred into a 40 mL glass vial. Glass equipment was used throughout the procedure, as the particles tend to adhere to plastic surfaces when cleaned with acetone. The IONPs were precipitated using a mixture of acetone and isopropyl alcohol (2 : 1 ratio) and magnetically separated for 10 minutes. The solvent was removed, and the particles were washed three times with 5–10 mL of acetone. The washed particles were then air-dried after the final wash. Subsequently, 5 mL of TMAH solution was added to the dried IONPs. The mixture was sonicated for 10 minutes to ensure uniform dispersion. The particles were then placed on a shaking table and shaken at 300 rpm for 2 hours. Following this, the particles were washed three times with 3 mL of Milli-Q water. Finally, the phase-transferred particles were dispersed in 10 mL Milli-Q water and stored in a refrigerator.^19^

#### 2.3.5 Characterization

##### 2.3.5.1 Size and morphology

A Hitachi SU9000 microscope was used to acquire electron micrographs of the IONPs in bright-field (BF) scanning transmission electron microscopy (STEM) mode at an accelerating voltage of 30 kV. Particle size and morphology were determined from the micrographs by manually measuring 200 particles per sample using ImageJ software.

Intensity-based hydrodynamic diameter (*H*_d_) and zeta potential (*ζ*-potential) of phase-transferred IONPs were measured using a Litesizer 500 (Anton Paar). Standard deviations were calculated from three independent measurements for each experimental condition. To determine the particle size based on sedimentation velocity, a multisample analytical centrifuge, LUMiSizer (L.U.M. GmbH, Berlin, Germany), was utilized.

##### 2.3.5.2 Crystal structure

The crystal structure of the IONPs was analyzed using a Bruker D8 Advance Da Vinci X-ray diffractometer (XRD). A few drops of the IONP suspension were deposited onto a silica chip, dried under ambient conditions, and covered with Kapton film prior to measurements. XRD patterns were collected using Cu Kα radiation (*λ* = 1.5406 Å) over a 2*θ* range of 20°–80° with a step size of 0.045°. Background subtraction and preliminary peak analysis were performed using DIFFRAC.EVA software in combination with the PDF-5+ database.

Lattice parameters and crystallite sizes were determined by Pawley refinement using TOPAS software. All diffraction patterns were indexed to a cubic spinel structure corresponding to the *Fd*3̄*m* space group (no. 227). Peak profiles were fitted using a pseudo-Voigt function to account for both Gaussian and Lorentzian contributions to peak broadening. Representative Pawley-fitted XRD diffractograms for as-synthesized and phase-transferred IONPs are provided in the SI Fig. S1 and S2.

Crystallite sizes were calculated using the Scherrer equation:1
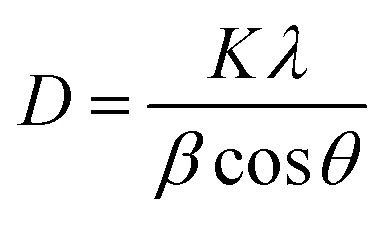
where *D* is the crystallite size, *K* is the shape factor (0.9 for spherical particles), *λ* is the X-ray wavelength, *β* is the full width at half maximum (FWHM) of the diffraction peak (corrected for instrumental broadening), and *θ* is the Bragg angle. Instrumental resolution and peak-shape parameters refined during the Pawley fitting were incorporated into the calculations to ensure accurate size estimation.

Two IONP samples were characterized by transmission electron microscopy (TEM). A JEOL ARM200F microscope integrated with spherical aberration correctors was operated at 200 kV. High-resolution TEM (HR-TEM) images were collected using a Gatan Rio 1816 CMOS 4k camera. Selected area electron diffraction (SAED) patterns were acquired on a MerlinEM direct electron detector (Quantum Detectors, quad, 512 × 512 px). The SAED patterns were processed using the open-source python libraries hyperspy^20^ and pyXem^21^ (further explained in the SI, Section A.2).

##### 2.3.5.3 Magnetic properties

Saturation magnetization (*M*_s_) measurements were conducted at 300 and 5 K using a MicroMag™ 3900 vibrating sample magnetometer (VSM) under a magnetic field of 1 T with field increments of 0.02 T and commercial quantum design physical property measurement system (PPMS) with max field of 5 T for low temperature measurements. After the VSM analysis, gel capsules containing dried IONPs were digested in 69% nitric acid using a Berghof SpeedWave microwave digestion system. The iron content in the digested samples was quantified using an Agilent Microwave Plasma Atomic Emission Spectroscopy (MP-AES) 4210 optical emission spectrometer (details in SI Section A.3). After Fe quantification, it is important to mention that all the *M*_s_ values reported in this manuscript were normalized to Fe fraction.

##### 2.3.5.4 Surface chemistry

Surface functional groups were characterized using attenuated total reflection Fourier-transform infrared (ATR-FTIR) spectroscopy. Measurements were performed on dried samples in transmittance mode under vacuum (3 mbar) using a Bruker Vertex 80v FTIR spectrometer at a resolution of 4 cm^−1^, with 100 scans accumulated and analyzed using OPUS software.

##### 2.3.5.5 Magnetic resonance relaxivities

MR relaxivities of selected IONPs that were phase transferred with the oxidative cleavage method were measured using a preclinical 7 T MR scanner (Bruker BioSpec) with a quadrature volume coil. The IONPs were dispersed in water in an Fe concentration range of 0.06–0.9 mM, loaded into NMR tubes, which were fixed in a tube containing water, to reduce susceptibility artifacts (see Fig. 8a for the resulting MR image from this phantom). *T*_1_ mapping was performed using a RARE sequence with variable repetition times (VTR). The imaging parameters were: TE = 14 ms; TR = 25, 50, 100, 250, 500, 1000, 2500, 3500 ms; in-plane resolution = 0.2 × 0.2 mm^2^; slice thickness = 2 mm. *T*_2_ mapping was acquired with the same spatial resolution and imaging volume, using a Multi-Slice Multi-Echo (MSME) sequence with 25 echo times ranging from 8 to 200 ms and a TR of 2000 ms. The obtained relaxation rates 1/*T*_1_ and 1/*T*_2_ were plotted as a function of Fe concentration, and the slope of these graphs was determined and represents the *T*_1_ and *T*_2_ relaxivities, *r*_1_ and *r*_2_, respectively.

## 3 Results and discussion

### 3.1 Synthesis of IONPs by thermal decomposition method

#### 3.1.1 Iron pentacarbonyl

Using Fe(CO)_5_ as the iron precursor, two distinct thermal decomposition syntheses were performed: one employing DDAB and the other using OAm as the surface ligand, as detailed in Sections 2.2.1 and 2.2.2, respectively.

In the DDAB-based synthesis, we used 0.8 mmol of DDAB as a capping agent, which resulted in the formation of spherical nanoparticles with fused morphology. Moreover, particles exhibited poor colloidal stability and rapidly sedimented upon storage in toluene. Size analysis from electron micrograph revealed that the particles had an average diameter of 8 ± 1 nm which is notably smaller than the 12 ± 1 nm reported in the reference study using the same DDAB concentration (Fig. 1a).^13^

**Fig. 1:**
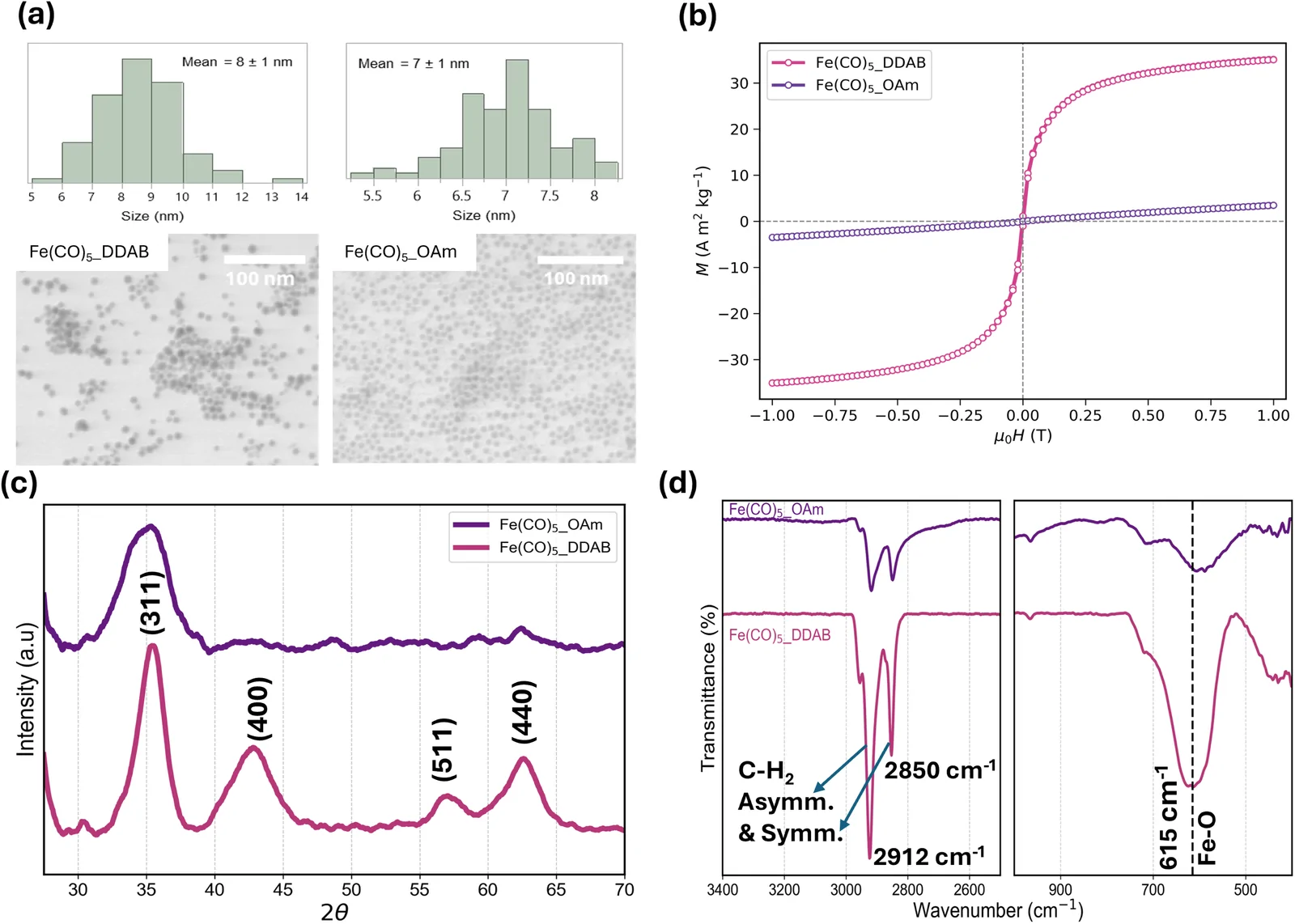
(a) Representative STEM images of IONPs synthesized using Fe(CO)_5_ as the precursor, with DDAB (left) and OAm (right) as surface ligands. Scale bars: 100 nm. Size-distribution histograms are displayed above each image. (b) Magnetic hysteresis curves showing differences in *M*_s_ normalized to Fe content. (c) XRD patterns confirm spinel iron oxide as the predominant phase. (d) FTIR spectra showing Fe–O vibrations and C–H stretching bands indicative of iron oxide and organic ligands, respectively.

The *M*_s_ of the synthesized nanoparticles was measured by VSM at 300 K (Fig. 1b). Magnetization values were normalized to the iron content using MP-AES. Under this normalization, the *M*_s_ was 35 A m^2^ kg^−1^ at 300 K. This reduced magnetic response is attributed to poor crystallinity, interfacial disorder within the nanoparticles, and most importantly due to the presence of undesired phases of Fe_*x*_O_*y*_ such as wüstite phase.

Scherrer analysis of the XRD data yields an average crystallite size of approximately 4.3 nm, substantially smaller than the particle diameter determined from STEM 8 ± 1 nm. A summary of different structural properties of IONPs is mentioned in Table 2. This disparity indicates that the particles are not fully coherent single crystals but instead consist of poorly crystalline or multi-domain structures. Such structural disorder, combined with the high surface-to-volume ratio at this size scale, is expected to promote spin canting and magnetically disordered surface layers, thereby significantly suppressing the net magnetization.^22^

**Table 2:** Summary of structural and magnetic properties of IONPs synthesized using different precursors and methods. *D*_STEM_ and *D*_XRD_ are in nm; *M*_s_ in A m^2^ kg^−1^ (of Fe) and lattice constants in Å. Phases are assigned from XRD and FTIR. No phase assignment is provided for the Fe(CO)_5_-derived sample because of extensive peak broadening in the XRD pattern; however, its calculated lattice constant is close to that of γ-Fe_2_O_3_

Sample	*D*_STEM_	*D*_XRD_	*M*_s_	Phase	Lattice constant (Å)
Fe(CO)_5__DDAB	8 ± 1	4.3	35	—	8.347
Fe(CO)_5__OAm	7 ± 1	1.8	3.5	—	8.344
FeOl_OA	11 ± 1	6.7	79	Fe_3_O_4_/γ-Fe_2_O_3_	8.353
FeOl_NaOl	15 ± 3	13.5	88	Fe_3_O_4_/γ-Fe_2_O_3_	8.374
Fe(acac)_3__OAm	7 ± 1	5.3	85	Fe_3_O_4_	8.380
Fe(acac)_3__OA/OAm/HDD	13 ± 2	13.8	112	Fe_3_O_4_	8.390

Analysis of the XRD pattern (Fig. 1c) shows broad diffraction features that fall within the 2*θ* ranges expected for spinel iron oxides, such as Fe_3_O_4_ and/or γ-Fe_2_O_3_. Owing to the substantial peak broadening, however, definitive phase identification is not possible, and the presence of additional overlapping reflections from other phases cannot be ruled out. The dominant diffraction peaks are located near the (311), (400), (511), and (440) reflections which are characteristic of spinel iron oxides. This contrasts with the reference study of Singh *et al.*,^13^ which reported diffraction peaks at (110), (200), and (211) planes corresponding to body-centered cubic (bcc) metallic iron and did not observe reflections associated with iron oxide phases, despite employing an otherwise identical synthetic protocol. The discrepancy between the present XRD results and the reference study which reported only bcc-Fe reflections can be rationalized by two factors. First, the extremely small crystallite size and limited shell thickness of the nanoparticles can lead to significant peak broadening, rendering the reflections difficult to resolve by laboratory XRD.^23^ Second, nanoscale metallic iron is highly susceptible to oxidation, and even brief exposure to oxygen during cooling, cleaning, or sample preparation can result in partial or complete transformation to spinel iron oxide phases.

Since Fe_3_O_4_ and γ-Fe_2_O_3_ share similar spinel structures and overlapping peaks, distinguishing them solely by XRD is challenging. To address this, we applied Pawley fitting (details in SI Section A.1) to refine the lattice parameter and compared it with stoichiometric values for Fe_3_O_4_ and γ-Fe_2_O_3_. The refined lattice parameter was 8.347 Å, closely matching γ-Fe_2_O_3_ (8.346 Å; JCPDS 39-1346),^24,25^ suggesting that the sample predominantly consists of γ-Fe_2_O_3_.

FTIR spectra (Fig. 1d) further supported the presence of γ-Fe_2_O_3_. Typically, Fe_3_O_4_ exhibits a broad Fe–O peak at 580–590 cm^−1^ with a shoulder near 700 cm^−1^, whereas γ-Fe_2_O_3_ presents multiple peaks in the 800–400 cm^−1^ range.^26^ In our sample, a broadened Fe–O band centered at ∼615 cm^−1^ is characteristic of γ-Fe_2_O_3_ or a defective spinel closely related to it.^27^ Intense C–H stretching bands (2800–2950 cm^−1^) indicate an organic (DDAB) layer on the nanoparticle surface.

In a second synthesis using Fe(CO)_5_ as the precursor, we employed OAm as a surfactant, following an adapted protocol from Peng *et al.*^14^ Here, OAm was increased to 2.3 mmol and the solvent (octadecene) to 50 mL. The resulting particles were spherical, core–shell, and 7 ± 1 nm in size (Fig. 1a and SI S8).

The nanoparticles exhibited a very low *M*_s_ of 3.5 A m^2^ kg^−1^ at 300 K (Fig. 1b). Low-temperature magnetization at 5 K (Fig. S3) also revealed a pronounced reduction in *M*_s_ (∼50% lower relative to other samples). Interestingly, these nanoparticles showed a comparable *M*_s_ reduction but an elevated coercive field *H*_c_ (Table S2), which suggests an increase in magnetic anisotropy.^28^ We attribute the reduced magnetic performance primarily to the amorphous shell formed during synthesis, which probably enhances spin disorder and anisotropy. However, the possibility of the presence of other non/less magnetic phases of Fe_*x*_O_*y*_ cannot be neglected.

Nanoparticles prepared through the OAm route lacked adequate crystallinity. STEM images revealed an amorphous shell around a crystalline core (SI Fig. S8). XRD pattern (Fig. 1c) showed broad reflections at ∼35°, that is indexed to (311) lattice plane of Fe_3_O_4_ or γ-Fe_2_O_3_. However, due to the broadness of the peak, the presence of other phases of Fe_*x*_O_*y*_ cannot be neglected. The calculated lattice parameter was 8.344 Å, close to γ-Fe_2_O_3_ (8.346 Å).^24,25^ which aligns with Williams *et al.*^29^ analysis who identified γ-Fe_2_O_3_ as dominant phase in these particles by Mössbauer spectroscopy. FTIR showed a broadened Fe–O band at 615–630 cm^−1^ that is a characteristic of γ-Fe_2_O_3_ or defective spinels.^27^

#### 3.1.2 Iron oleate

Using Fe(Ol) as the iron precursor, spherical and cubic IONPs were synthesized following the method explained in Sections 2.2.5 and 2.2.6, respectively. The protocols differ primarily in the surface ligand used during thermal decomposition: OA promotes spherical morphology, whereas NaOl favors cubic shapes.

The Fe(Ol)_OA route for spherical IONPs is well established since the work of Park *et al.*^3^ and allows tuning of size and morphology *via* the OA amount, heating rate, reflux temperature, and solvent composition.^30^ In our case, repeated syntheses have consistently demonstrated the reproducibility of this process in terms of both size distribution and shape of the particles (Fig. 2a). The average size of spherical particles measured from electron microscopy images is 11 ± 1 nm after counting 200 particles across multiple representative STEM images. Introducing NaOl in the Fe(Ol) synthesis promoted anisotropic growth and predominantly cubic particles with a diameter of 15 ± 3 nm. Scherrer analysis of the XRD data yielded average crystallite sizes of approximately 6.7 nm for spherical and 13.5 nm for cubic IONPs, both of which are smaller than the corresponding particle diameters determined by STEM (Table 2).

**Fig. 2:**
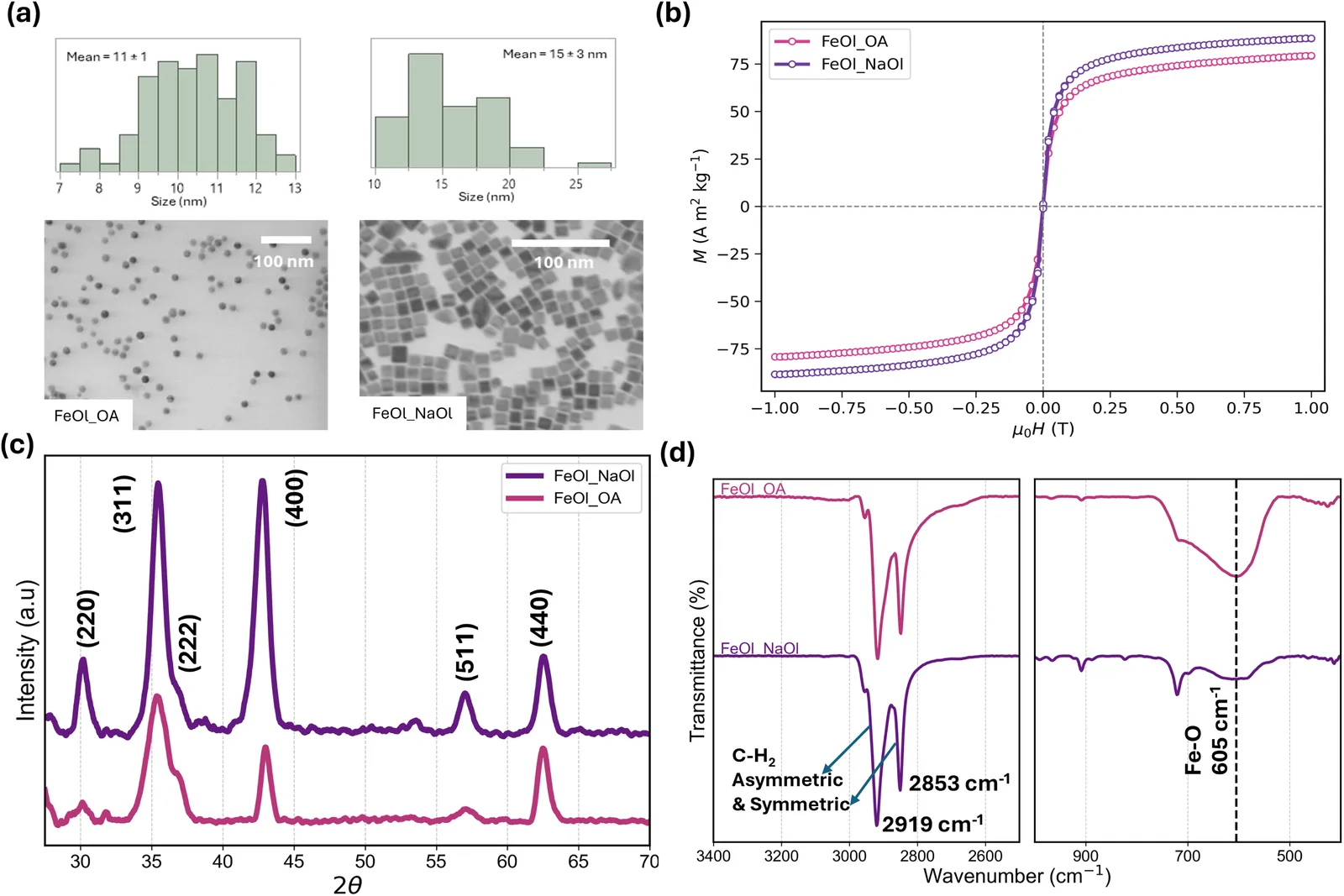
(a) Representative STEM images of IONPs synthesized using Fe(Ol) with OA (left) and NaOl (right) as ligands. Scale bars: 100 nm. histograms above each image show size distributions. (b) Magnetic hysteresis curves showing differences in magnetization of both samples, normalized to Fe content. (c) XRD patterns confirm spinel iron oxide as the predominant phase. (d) FTIR spectra of the IONPs.

The coefficient of variation (CV = *σ*/*µ*) is 0.09 for spheres and 0.20 for cubes. The larger CV for cubes reflects the intrinsic sensitivity of faceted growth (edges/corners) to small kinetic fluctuations and the more complex coordination introduced by sodium carboxylates. In addition, batch-to-batch variability of Fe(Ol) precursors is known to affect reactivity and nucleation/growth kinetics,^31,32^ which likely contributed to the broader dispersion in the Fe(Ol)_NaOl samples.

Room-temperature (300 K) magnetization curves (Fig. 2b) show superparamagnetic behavior for both spherical and cubic nanoparticles, characterized by negligible coercivity and remanence. The *M*_s_ values were approximately 79 A m^2^ kg^−1^ and 88 A m^2^ kg^−1^, respectively, when normalized to the iron content. These values are substantially higher than those obtained for the Fe(CO)_5_-derived nanoparticles synthesized in this work, indicating improved phase development and crystallinity associated with the Fe(Ol) route.

XRD patterns of both the spherical and cubic Fe(Ol)-derived nanoparticles (Fig. 2c) exhibit reflections characteristic of inverse-spinel iron oxides, with prominent peaks indexed to the (220), (311), (222), (400), (511), and (440) planes. However, due to the peak broadness, the presence of Wüstite (FeO) phase in these samples, particularly in spherical IONPs is possible, as we have reported in our previous work.^30^ Pawley-refined lattice parameters were 8.353 Å for the spherical nanoparticles and 8.374 Å for the cubic nanoparticles. These values fall between those of bulk magnetite, Fe_3_O_4_ (8.396 Å), and maghemite, γ-Fe_2_O_3_ (8.346 Å), suggesting mixed-phase compositions rather than a single phase.

FTIR spectra (Fig. 2d) further support this interpretation. Both samples display a broad Fe–O stretching feature with a minima near ∼605 cm^−1^, intermediate between the typical Fe–O bands of Fe_3_O_4_ (∼570 cm^−1^) and γ-Fe_2_O_3_ (∼630 cm^−1^),^27^ which is an indication of the coexistance of both phases of iron oxide at the nanoscale.

The relative improvement in magnetic properties of cubic and spherical IONPs compared to Fe(CO)_5_-derived IONPs could be explained by their differences in XRD patterns. XRD analysis reveals sharper and more intense reflections for the Fe(Ol)-derived samples compared to the broader, less defined diffraction features observed for Fe(CO)_5_-derived nanoparticles. Furthermore, the difference between particle diameters measured by STEM and crystallite sizes estimated from XRD is smaller for the Fe(Ol)-derived samples, which also suggests a relatively higher degree of structural coherence and reduced interfacial disorder.

Nevertheless, the magnetization values remain sub-optimal due to surface disorder. In both samples, particle diameters from STEM exceed crystallite sizes from XRD (*D*_STEM_ > *D*_XRD_), suggesting the presence of an amorphous/poorly crystalline surface layer around a more ordered core. Such heterogeneity promotes spin canting and magnetically “dead” surface regions, which reduce the effective magnetization relative to the bulk materials.^33^

#### 3.1.3 Iron acetylacetonate

Using Fe(acac)_3_ as the iron precursor, two thermal decomposition syntheses were carried out: one employing OAm as the sole surfactant (Section 2.2.3) and a second using a mixed-ligand system consisting of OAm, OA, and HDD (Section 2.2.4).

In the OAm-only synthesis, thermal decomposition of Fe(acac)_3_ in the presence of excess OAm yielded predominantly irregular, quasi-spherical nanoparticles with an average diameter of 7 ± 1 nm (Fig. 3a). Magnetic measurement performed at 300 K (Fig. 3b) yielded *M*_s_ values of 85 A m^2^ kg^−1^ when normalized to Fe content.

**Fig. 3:**
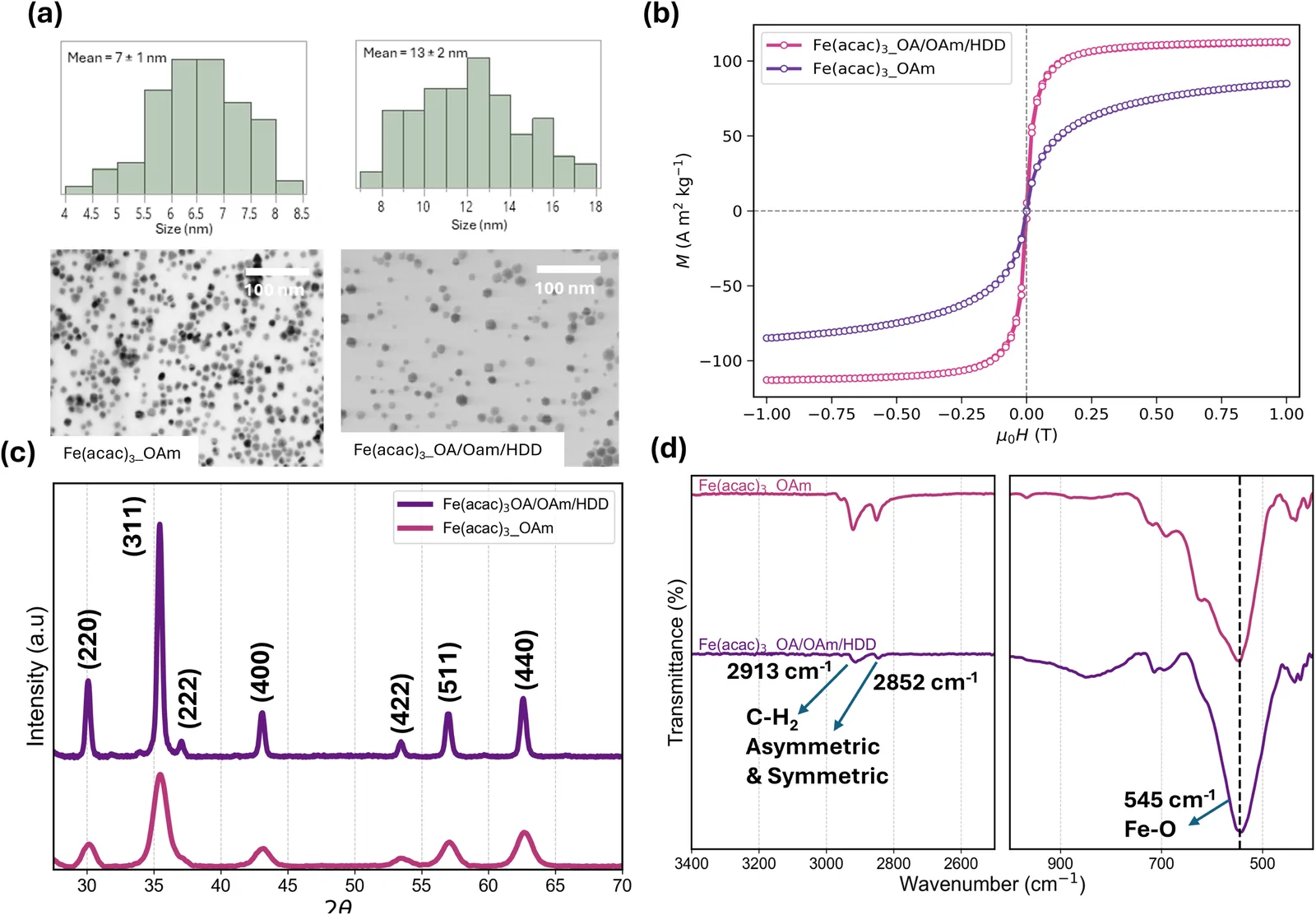
(a) Representative STEM images of IONPs synthesized using Fe(acac)_3_ with OAm (left) and OA/OAm/HDD (right). Scale bars: 100 nm. Histograms show size distributions. (b) Magnetic hysteresis curves comparing magnetization of samples, normalized to Fe content. (c) XRD patterns confirm spinel iron oxide as the predominant phase. (d) FTIR spectra of the IONPs.

During the reaction, a large excess of OAm provides a strongly reducing and coordinating environment that facilitates precursor decomposition and rapid nucleation. Although particle size can, in principle, be tuned by adjusting the benzyl ether : OAm ratio,^15^ reproducible size and shape control proved challenging in practice. This limitation arises from the thermal instability of benzyl ether at the high temperatures required for Fe(acac)_3_ decomposition.^30^ Moreover, at elevated temperatures, benzyl ether undergoes partial decomposition into more polar species (*e.g.*, benzaldehyde), leading to a gradual increase in the polarity of the reaction medium. This change weakens the solvophobic interactions between the solvent and aliphatic ligand molecules at the nanoparticle surface. Consequently, this leads to the loss of growth control and leads to particles of irregular morphology.^34^

XRD analysis (Fig. 3c) revealed a series of reflections indexed to the (220), (311), (222), (400), (422), (511), and (440) planes, consistent with a cubic spinel iron oxide structure. The refined lattice parameter (*a* = 8.380 Å) closely matches that of magnetite (Fe_3_O_4_; *a* = 8.396 Å, JCPDS 19-0629),^24,25^ supporting the formation of a predominantly Fe_3_O_4_ phase. Complementary FTIR spectroscopy (Fig. 3d) shows a well-defined Fe–O stretching vibration centered at ∼545 cm^−1^, which lies within the characteristic range reported for magnetite and that further supports the evidence of magnetite as a dominant phase in these IONPs.^35,36^

Despite strong structural evidence for a predominantly magnetite phase, magnetization values remain significantly lower, and are comparable to those obtained for Fe(Ol)-derived nanoparticles exhibiting mixed magnetite/maghemite character. This reduction in magnetization is therefore attributed primarily to nanoscale structural effects rather than phase composition alone. A key factor is the limited crystallinity and pronounced surface disorder inherent to ultrasmall nanoparticles. The average particle diameter determined by STEM (*D*_STEM_ = 7 nm) exceeds the crystallite size extracted from Scherrer analysis of the XRD data (*D*_XRD_ = 5.3 nm), indicating the presence of a structurally disordered or amorphous surface layer of approximately 1–2 nm thick. The observed discrepancy between *D*_STEM_ and *D*_XRD_ may imply here the presence of spin-disordered shell. This is consistent with well-established observations in the literature.^22,33^

The second synthesis, adapted from Yang *et al.*,^16^ which employed a mixed-ligand system comprising OAm, OA, and HDD. In contrast to the OAm-only synthesis, this approach yielded a heterogeneous population of nanoparticles, including cubes and truncated octahedra. The absence of a single, well-defined morphology indicates that precise shape control is not achieved under these conditions. The mean size of the IONPs measured from electron micrographs is 13 ± 2 nm. A representative electron micrograph along with the size distribution of particles in shown in Fig. 3a.

The origin of this shape heterogeneity is likely multifactorial. One contributing factor may be the thermal instability of benzyl ether at the elevated temperatures required for Fe(acac)_3_ decomposition. Partial decomposition of benzyl ether into benzaldehyde and benzyl benzoate has noticed to induce temperature fluctuations and alter the chemical environment during growth.^34^ In addition, oxygenated decomposition products can modify surface energies and preferentially stabilize specific crystal facets, particularly the (111) planes, suppressing ideal cubic growth and favoring truncated octahedral morphologies exposing mixed (100)/(110)/(111) facets.^37^

Magnetic measurements revealed a *M*_s_ of 112 A m^2^ kg^−1^ of Fe (Fig. 3b). At 5 K, the cubic Fe(acac)_3__OA/OAm/HDD nanoparticles exhibited the highest *M*_s_ among all samples investigated (Fig. S3). XRD analysis of the OA/OAm/HDD sample (Fig. 3c) revealed diffraction peaks characteristic of spinel iron oxide phases, indexed to the (220), (311), (222), (400), (422), (511), and (440) planes. Compared to the rest of synthesis, these reflections are markedly sharper which indicates enhanced crystallinity and larger coherent diffraction domains of synthesized IONPs. The refined lattice parameter was 8.390 Å which is in close agreement with stoichiometric Fe_3_O_4_ (8.396 Å).^24,25^ This confirms the formation of a well-ordered magnetite phase. Moreover, FTIR spectra (Fig. 3d) further corroborated this observation which is displaying a distinct Fe–O stretching band at ∼545 cm^−1^ that are consistent with magnetite, as mentioned in previous reports.^35,36^

This batch of IONPs has superior magnetic properties of all the particles synthesized, which is consistent with the high crystallinity of the nanoparticles and the effective suppression of surface spin disorder. This is evidenced by the good agreement between particle sizes obtained from STEM and XRD (Table 2). STEM analysis yielded an average particle diameter of 13 ± 2 nm for the OA/OAm/HDD sample, while Scherrer analysis of the XRD data gave a crystallite size of 13.8 nm. The close correspondence between these values indicates a negligible amorphous or structurally disordered surface layer, which could be the basis for the near-bulk magnetization observed in this system.

### 3.2 Overview of synthesis methods

Overall, Fe(CO)_5_-based syntheses produce the smallest particles but are limited by poor colloidal stability (often settling at the bottom of the vial during storage), limited crystallinity, and fused morphologies. Large discrepancies between STEM-derived particle size and XRD-derived crystallite size indicate structural disorders and largely amorphous character, which results in suppressed magnetization due to surface spin canting and interfacial defects.

Fe(acac)_3_-based routes offer greater tunability through ligand engineering but demand careful control of reaction conditions. Using OAm alone yields ultrasmall, predominantly Fe_3_O_4_ nanoparticles with reduced crystallinity and diminished magnetization due to a substantial disordered surface fraction. In contrast, incorporating OA and HDD largely enhances crystallinity, suppresses surface disorder, and produce nanoparticles with near-coherent crystalline domains and magnetization values. However, achieving consistent morphology with Fe(acac)_3_ remains challenging due to progressive benzyl ether degradation at elevated temperatures, which destabilizes the reaction. Limited stability of particles during a long term storage is also a concern for these particles.

In contrast, the FeOl-based route emerges as the most robust and reproducible platform, offering reliable control over size and morphology through ligand selection (OA *vs.* NaOl). FeOl-derived IONPs consistently exhibit narrow size distributions, relatively sharp XRD reflections, minimal disparity between STEM and XRD sizes, and high *M*_s_ values. We believe that this combination of structural coherence, magnetic performance, and synthetic robustness makes FeOl the most reliable precursor for producing IONPs specifically for biomedical applications.

To further contextualize these observations, Table 3 compares the TEM-derived particle sizes and identified crystalline phases obtained in this work with representative literature reports for similar synthesis routes. While the overall trends are consistent, noticeable differences in particle size are evident. For example, Fe(CO)_5_-based systems in the present study yield slightly smaller particles, whereas Fe(acac)_3_- and FeOl-based systems show moderate variations in size and phase identification. Such discrepancies are expected given the strong sensitivity of thermal decomposition syntheses to experimental conditions. Parameters including precursor purity (commercial *versus* home-synthesized), degree of hydration, aging of reagents, solvent purity, and even the positioning of the thermocouple within the reaction setup can influence the reaction. As a result, small variations in these factors can lead to measurable differences in particle size, crystallinity, and phase composition.

**Table 3:** Comparison of STEM size and identified crystalline phase between the current work and literature reports

Sample	Current work	Literature
Fe(CO)_5__DDAB	8 ± 1; γ-Fe_2_O_3_	12 ± 1; Fe@Fe_*x*_O_*y*_ (ref. 13)
Fe(CO)_5__OAm	7 ± 1; γ-Fe_2_O_3_	6.5; Fe@Fe_3_O_4_ (ref. 14)
FeOl_OA	11 ± 1; Fe_3_O_4_/γ-Fe_2_O_3_	12; Fe_3_O_4_/γ-Fe_2_O_3_ (ref. 3)
FeOl_NaOl	15 ± 3; Fe_3_O_4_/γ-Fe_2_O_3_	9–23; Fe_3_O_4_/γ-Fe_2_O_3_ (ref. 3 and 38)
Fe(acac)_3__OAm	7 ± 1; Fe_3_O_4_	7–10; Fe_3_O_4_ (ref. 15)
Fe(acac)_3__OA/OAm/HDD	13 ± 2; Fe_3_O_4_	15; Fe_3_O_4_ (ref. 16)

TEM characterization was performed to further study the morphology and crystal structure in one sample fabricated through the Fe(acac)_3_-based route and one sample made by the FeOl-based route. Fig. 4 includes SAED patterns and HR-TEM images of the Fe(acac)_3__OA/OAm/HDD ((a) and (d)) and FeOl_OA ((b) and (e)) samples. The SAED patterns show rings characteristic for multiple crystallites. The integrated profiles presented in Fig. 4c show strong correspondence to the XRD patterns, with Bragg peaks indexed with respect to the cubic magnetite structure. For both samples, HR-TEM imaging reveals mostly single crystal nanoparticles, with only a few displaying signs of multiple domains. Fig. 4d and e present HR-TEM images of a single nanoparticle from each sample, together with their fast Fourier transforms. The Fe(acac)_3__OA/OAm/HDD sample primarily contains faceted nanoparticles that typically appear hexagonal in projection. In the FeOl_OA sample, on the other hand, only spherical shapes are observed.

**Fig. 4:**
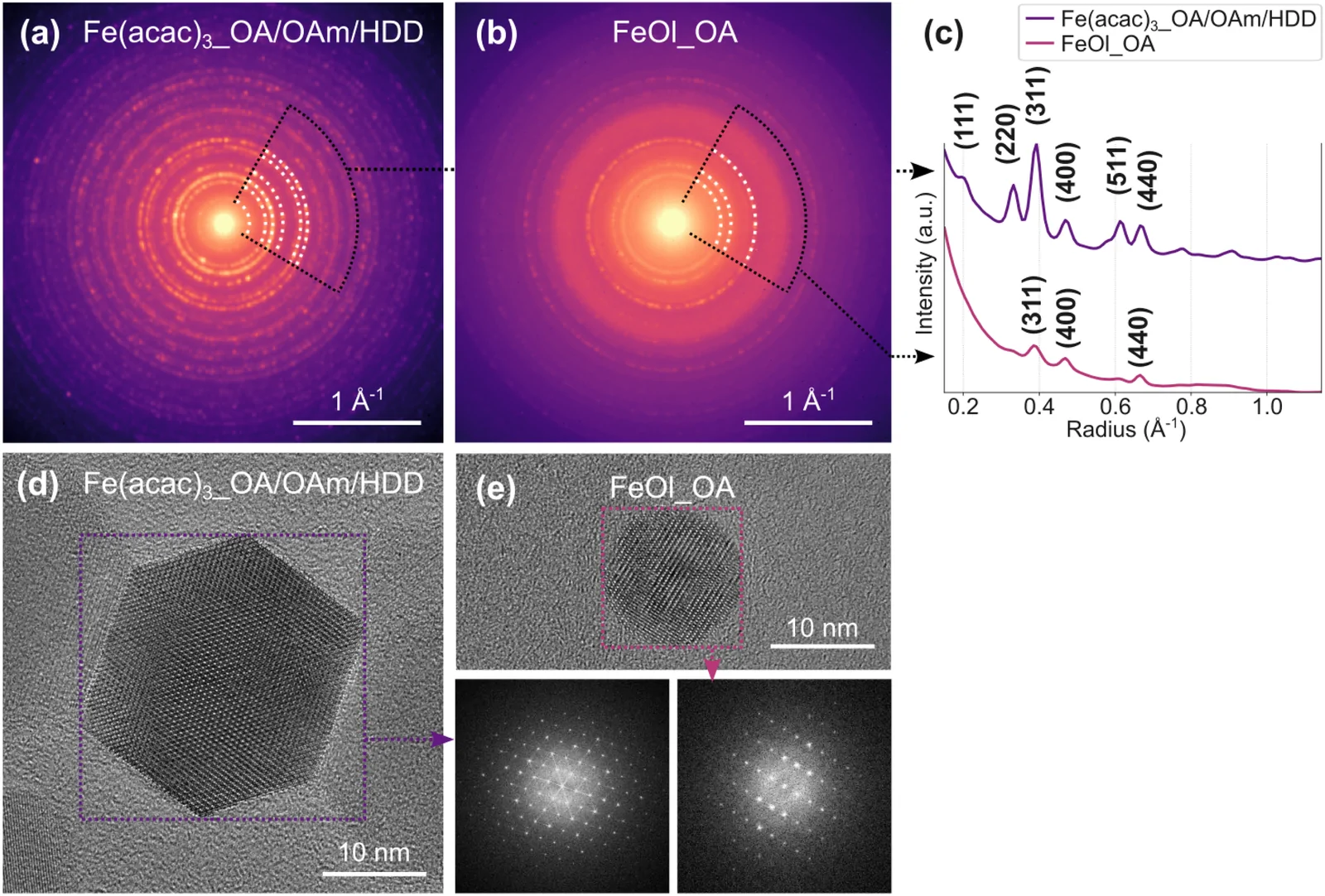
TEM characterization of Fe(acac)_3__OA/OAm/HDD and FeOl_OA. (a) and (b) SAED patterns (plotted on a logarithmic scale), with (c) corresponding integrated profiles showing Bragg peaks indexed according to the cubic magnetite structure. (d) and (e) HR-TEM images of single IONPs together with respective fast Fourier transforms.

### 3.3 Phase-transfer

Phase-transfer is a crucial step for IONPs synthesized by thermal decomposition, which converts hydrophobic particles into water-dispersible systems. The phase transfer process can substantially influence particle size, magnetic performance, stability, surface chemistry, and biological compatibility. In this section, the IONPs synthesized in the previous section were successfully phase-transferred using four representative strategies: (i) elimination of organic ligands *via* a base-bath-assisted method, (ii) oxidative cleavage of surface ligands, (iii) ligand exchange with sodium citrate (NaCit), and (iv) ligand exchange with tetramethylammonium hydroxide (TMAH). While all six IONP batches were phase-transferred and characterized, the discussion primarily focuses on Fe(acac)_3__OA/OAm/HDD and FeOl_OA nanoparticles, which serve as reference systems to explain how precursor chemistry and nanoparticle structure influence phase-transfer efficiency and post-transfer properties.

#### 3.3.1 Elimination of organic ligands through base-bath-assisted method

The base bath-assisted phase transfer procedure was adapted from the work of Vilas-Boas *et al.*,^18^ where they successfully performed phase transfer of oleate-capped IONPs synthesized *via* thermal decomposition. This method has drawn its inspiration from the alkaline base bath solutions used to eliminate organic contaminants and residues from various surfaces. The alkaline nature of the solution aids in emulsifying and saponifying organic materials, which simplifies their removal from surfaces. Therefore, it was hypothesized that a similar mechanism could be applied to remove organic ligands from the surfaces of nanoparticles by introducing a base solution to the IONPs. Using this method we successfully phase transferred all the IONPs types. Fig. S6 in SI visually demonstrates successful phase transfer of IONPs from the upper organic layer to the lower aqueous layer.

#### 3.3.2 Phase-transfer through oxidative cleavage of surface ligands

Oxidative cleavage was employed to phase-transfer IONPs stabilized by organic ligands containing unsaturated hydrocarbon chains. In this process, carbon–carbon double bonds present in unsaturated ligands, such as OA, are cleaved and converted to hydrophilic carboxyl groups on the IONP surface which enables the particles to be dispersible in water.^10^ Interestingly, oxidative cleavage was also effective for nanoparticles coated with ligands other than OA. While the precise mechanism in these cases remains less well understood, a plausible explanation is that the ligands employed in this study similarly contain unsaturated moieties and long hydrocarbon chains analogous to OA. These structural features are expected to undergo similar oxidative scission, generating polar surface groups that promote phase transfer.

#### 3.3.3 Phase-transfer through ligand exchange

Using this approach, we phase-transferred IONPs by two ligand-exchange routes. First, NaCit acted as the phase-transferring agent: its two symmetric and one asymmetric carboxylate groups effectively replace hydrocarbon chains of surface-bound ligands and bind the IONP surface *via* carboxylate coordination. We implemented a modified protocol based on Sharma *et al.*^17^ Whereas Sharma employed 2 h water-bath sonication, we used probe sonicator that effectively reduced the phase-transfer duration to 20 min.

Second, we used TMAH, which acts as an electrolyte to displace organic ligands from the nanoparticle surface and stabilize particles in aqueous media.^19^

#### 3.3.4 Characterization of physicochemical properties of phase-transferred IONPs

Fig. 5 shows photographic images of IONP dispersions before and after phase transfer using the oxidative cleavage method (corresponding images for the base-bath and ligand exchange method are provided in SI Fig. S6). As can be seen from the images, after phase-transfer, the nanoparticles are uniformly dispersed in the aqueous phase, with no visible residue remaining in the organic phase, which indicates an efficient and complete phase-transfer of IONPs without macroscopic aggregation. Importantly, the IONPs retained their magnetic responsiveness following phase-transfer, as demonstrated by their successful magnetic separation in Fig. 5d.

**Fig. 5:**
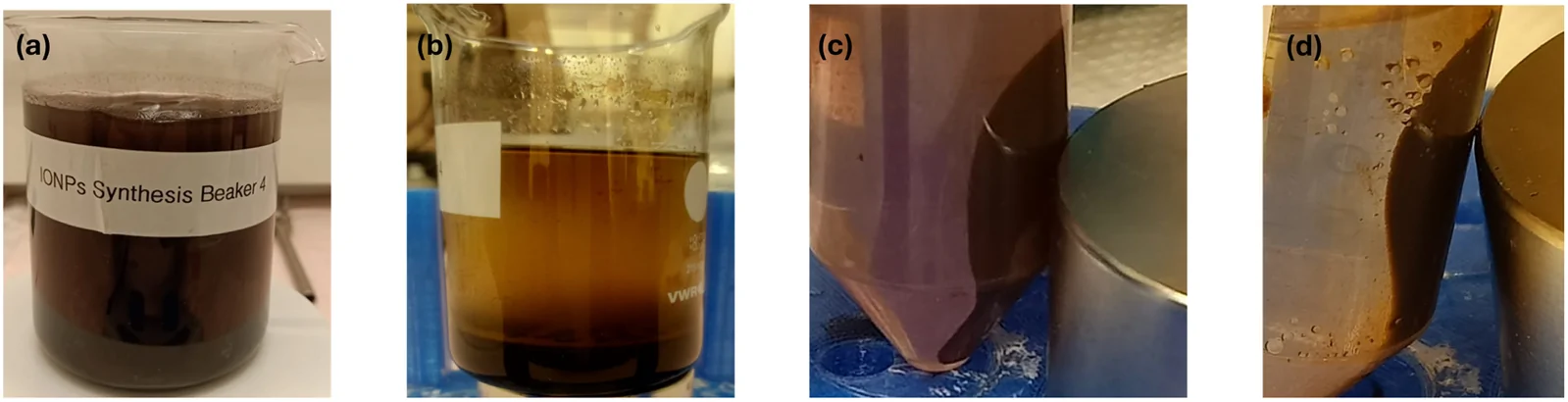
(a) Reaction mixture of FeOl_OA undergoing phase transfer in a biphasic system under magnetic stirring at room temperature. (b) Settling of Fe_3_O_4_ nanoparticles into the aqueous phase at the end of the reaction. (c) Magnetic separation of Fe_3_O_4_ nanoparticles from the turbid supernatant during the initial washing step. (d) Magnetic separation of Fe_3_O_4_ nanoparticles from the clear supernatant after final washing.

STEM images of FeOl_OA IONPs after phase-transferred by base bath, oxidative cleavage, and ligand exchange with NaCit and TMAH are shown in Fig. 6a–d; additional images for other IONPs are provided in SI Fig. S7. These images show that size and morphology remain largely unaffected by phase-transfer treatments.

**Fig. 6:**
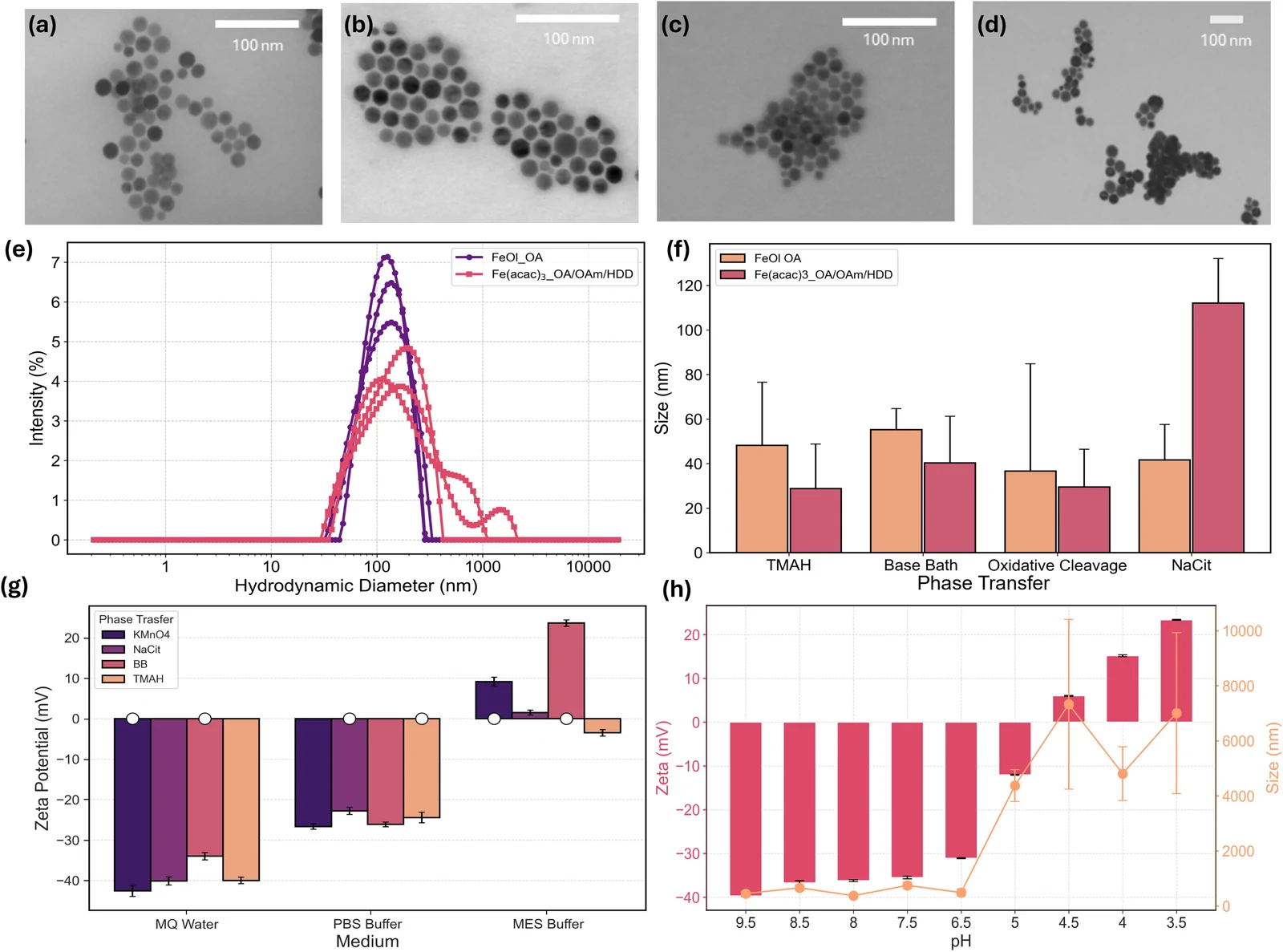
Representative STEM images of FeOl_OA IONPs phase-transferred using (a) oxidative cleavage, (b) base bath, (c) NaCit, and (d) TMAH methods. Scale bars: +100 nm. (e) Comparison of IONPs size measured using an AC after phase transferring IONPs with different methods. (f) *H*_d_ size distributions of phase-transferred FeOl_OA and Fe(acac)_3__OA/OAm/HDD IONPs by oxidative cleavage (DLS). (g) *ζ*-potential values of phase-transferred IONPs measured in MQ water, PBS buffer, and MES buffer. (h) *ζ*-potential values and *H*_d_ size of phase-transferred FeOl_OA (oxidative cleavage) *versus* pH.

The size of phase-transferred IONPs in aqueous media was evaluated using analytical centrifugation (AC) and dynamic light scattering (DLS). AC determines particle size based on sedimentation velocity and is, therefore, sensitive to the buoyant mass and compact aggregate size, while DLS measures *H*_d_ size from Brownian motion-induced light scattering.^39^ The AC-derived sizes of all phase-transferred IONPs are summarized in Table 4. Across all phase-transfer methods and particle types, AC sizes typically fell in the range of 50–100 nm, without a clear systematic dependence on the specific method employed. For instance, FeOl_OA and Fe(acac)_3__OA/OAm/HDD nanoparticles phase-transferred *via* oxidative cleavage exhibited AC sizes of 37 ± 20 nm and 29 ± 21 nm, respectively. In contrast, the same samples analyzed by DLS (Fig. 6e) displayed significantly larger apparent *H*_d_ sizes of 114 ± 2 nm and 145 ± 10 nm, respectively.

**Table 4:** Summary of *ζ*-potential values in Milli-Q water, PBS (pH 7.4), and MES buffer (pH 4.8), together with median particle size and standard deviation (Std) determined by AC for IONPs following different phase-transfer strategies

Phase transfer	IONPs	MQ water (mV)	PBS buffer (mV)	MES buffer (mV)	Median size (nm)
TMAH (OH^−^)	FeOl_OA	−47.9 ± 0.9	−24.4 ± 1.2	−9 ± 0.9	48 ± 28
	FeOl_NaOl	−54.5 ± 0.8	−34.9 ± 1.2	−12.1 ± 1	30 ± 21
	Fe(CO)_5__OAm	−36.7 ± 1.1	−24.6 ± 0.4	11.3 ± 1	41 ± 20
	Fe(CO)_5__DDAB	−30.2 ± 1.3	−24.6 ± 0.8	15.6 ± 0.6	37 ± 19
	Fe(acac)_3__OAm	−29.8 ± 0.8	−25.8 ± 0.3	20.4 ± 0.8	50 ± 26
	Fe(acac)_3__OA/OAm/HDD	−32.1 ± 0.6	−24.5 ± 0.7	2 ± 0.6	29 ± 9
Base bath(OH^−^)	FeOl_OA	−35.8 ± 0.9	−25.2 ± 0.9	18.8 ± 0.6	55 ± 48
	FeOl_NaOl	−35.7 ± 0.6	−21 ± 1	6.5 ± 0.8	43 ± 20
	Fe(CO)_5__OAm	−31.5 ± 0.8	−27.5 ± 1	10.4 ± 0.6	43 ± 17
	Fe(CO)_5__DDAB	−33 ± 0.8	−27.2 ± 0.7	11.2 ± 1.4	55 ± 36
	Fe(acac)_3__OAm	−28.3 ± 1.1	−29.7 ± 1.5	26.4 ± 1.6	101 ± 53
	Fe(acac)_3__OA/OAm/HDD	−32.2 ± 0.6	−27.1 ± 1.4	28.4 ± 1.4	40 ± 16
Oxd. cleavage (COO^−^)	FeOl_OA	−41.7 ± 1.4	−30.5 ± 0.8	−5.1 ± 0.7	37 ± 20
	FeOl_NaOl	−38 ± 0.6	−28 ± 0.3	−20.6 ± 0.8	43 ± 13
	Fe(CO)_5__OAm	−36.5 ± 0.6	−27.5 ± 1	23.4 ± 0.9	41 ± 20
	Fe(acac)_3__OAm	−28.3 ± 1.1	−26.2 ± 0.7	−9.9 ± 0.4	54 ± 36
	Fe(acac)_3__OA/OAm/HDD	−43.4 ± 0.7	−22.8 ± 0.8	23.3 ± 1.1	29 ± 21
NaCit(COO^−^)	FeOl_OA	−46.1 ± 1.1	−28.1 ± 1.3	1.8 ± 0.8	42 ± 17
	FeOl_NaOl	−44.1 ± 0.76	−26.4 ± 0.8	2.1 ± 1.1	31 ± 16
	Fe(CO)_5__OAm	−32.1 ± 1.1	−23.6 ± 0.9	2.1 ± 1	52 ± 43
	Fe(CO)_5__DDAB	−41.1 ± 0.7	−24.8 ± 0.9	3.4 ± 0.9	30 ± 25
	Fe(acac)_3__OAm	−43.8 ± 0.4	−26.1 ± 1.3	2.5 ± 1	93 ± 44
	Fe(acac)_3__OA/OAm/HDD	−34.1 ± 1	−17.5 ± 0.8	1.2 ± 0.9	112 ± 20

While no single phase-transfer strategy consistently yielded the smallest particle sizes across all six IONP systems, AC-derived sizes were systematically smaller than the corresponding DLS values. This discrepancy is expected as AC primarily reflects the size of the dense magnetic core or compact aggregates, whereas DLS measures the hydrodynamic diameter derived from Brownian motion-induced light scattering and includes contributions from surface coatings and solvation layers.^39^ The evolution of AC-derived sizes across four different phase transfer methods tested for FeOl_OA and Fe(acac)_3__OA/OAm/HDD IONPs is shown in Fig. 6f.

To quantitatively assess colloidal stability, *ζ*-potential measurements were performed, with values summarized in Table 4. Similar to the size measurements, no clear trend with respect to the phase-transfer method was observed. In Milli-Q water, *ζ*-potential values ranged from approximately −30 to −45 mV, exceeding the commonly accepted threshold for electrostatically stabilized colloids (∼−30 mV).^40,41^ These results demonstrate that all four phase-transfer approaches are capable of producing stable, water-dispersible IONPs with comparable colloidal properties. Since biomedical applications are a central motivation of this work, the colloidal stability of phase-transferred IONPs was further evaluated in biologically relevant media, namely phosphate-buffered saline (PBS, pH 7.4) and MES buffer (pH 4.8). PBS is routinely used in cell culture and biological assays, while MES is commonly employed during carbodiimide-mediated coupling reactions.^42^Fig. 6g summarizes the *ζ*-potential values of representative FeOl_OA and Fe(acac)_3__OA/OAm/HDD IONPs measured in Milli-Q water, PBS, and MES buffer. In Milli-Q water, both systems exhibited *ζ*-potential values of approximately −35 mV, which is an indication of good electrostatic stabilization. Upon transfer to PBS, the magnitude of the *ζ*-potential decreases, which is followed by the further reduction in MES buffer (pH 4.8), suggesting diminished colloidal stability under acidic conditions. As the phase-transfer experiments were performed from fixed IONP input masses rather than matched Fe concentrations (Section 2.3, SI Table S5), the DLS and *ζ*-potential results should be interpreted as comparisons under nominally similar preparation conditions rather than at strictly Fe-normalized concentrations.

To further evaluate the stability of IONPs, the pH of FeOl_OA IONPs dispersion phase transferred by oxidative cleavage method was varied from 9.5 to 3.5 in Milli-Q water, and both *H*_d_ Size and *ζ*-potential values were measured. Upon changing the pH, as shown in Fig. 6h, the nanoparticles remain highly stable under alkaline conditions, whereas progressive acidification leads to a sharp decrease in *ζ*-potential values accompanied by a pronounced increase in *H*_d_ size. Notably, at pH ∼ 5, the onset of aggregation becomes evident. This behavior can be linked to protonation and partial neutralization of surface carboxylate and hydroxyl groups at low pH, which reduces electrostatic repulsion and promotes interparticle attraction.^43^

Although phase-transferred nanoparticles exhibit appropriate size and *ζ*-potential for biomedical use, their crystallinity and magnetization vary. These differences can impact application suitability. Fig. 7a shows distinct trends by precursor: Fe(acac)_3__OA/OAm/HDD (as-synthesized and phase-transferred) retains *M*_s_ ∼ 110 A m^2^ kg^−1^ at 300 K, whereas FeOl_OA samples are ∼80 A m^2^ kg^−1^ of Fe. Thus, Fe(acac)_3__OA/OAm/HDD-derived nanoparticles are favored for applications requiring strong magnetization. Interestingly, all phase-transfer strategies preserved the magnetic properties of as-synthesized IONPs. None of the four phase transfer methods caused considerable degradation in *M*_s_ values. This robustness indicates that any of the evaluated phase-transfer routes can be used when retaining magnetic performance is the primary requirement.

**Fig. 7:**
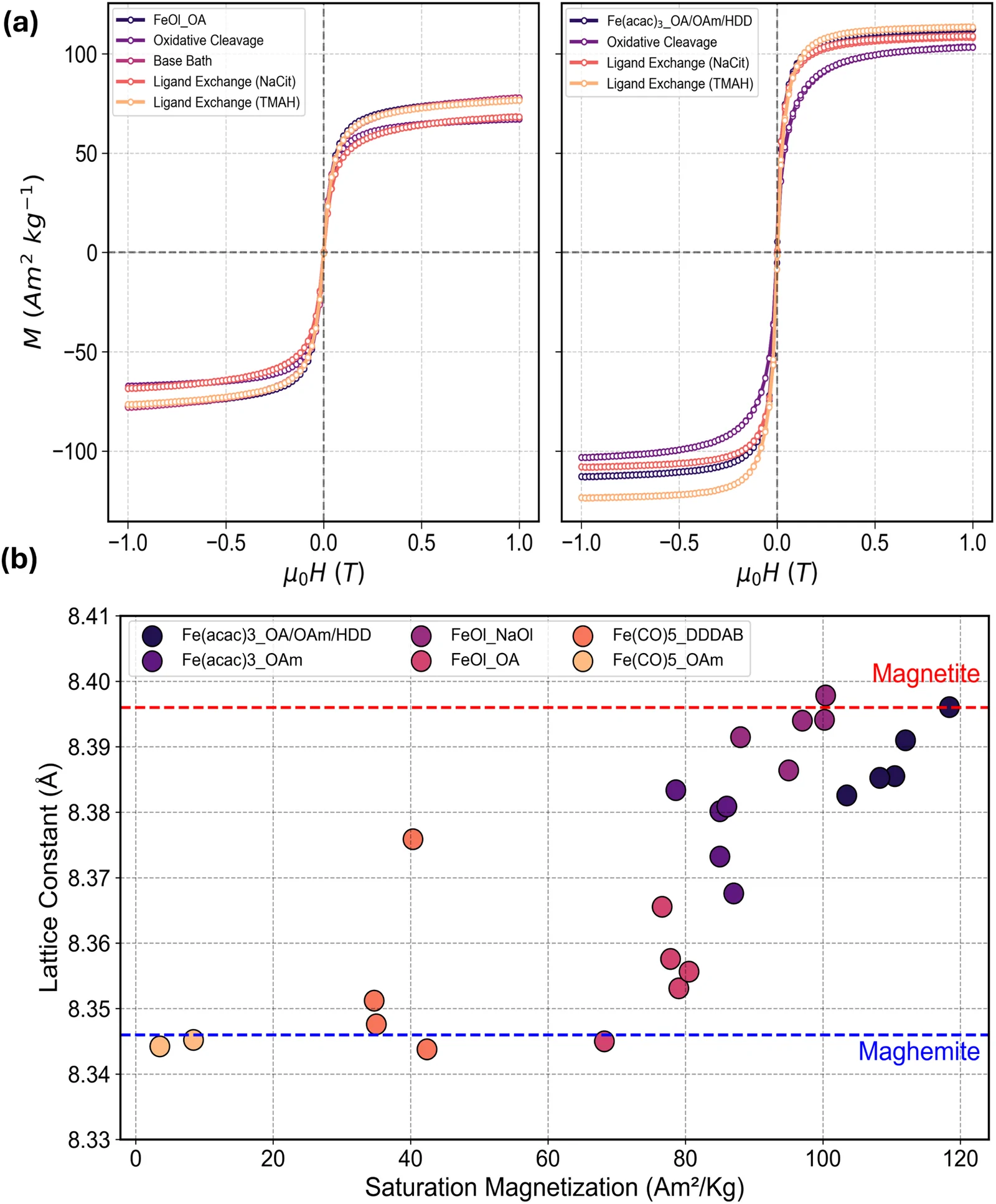
(a) Magnetization curves (*M vs. H*) normalized to Fe content for FeOl_OA (left) and Fe(acac)_3__OA/OAm/HDD (right) under different phase-transfer conditions, showing variations in magnetic properties. (b) Correlation between *M*_s_ and lattice parameter; reference bands indicate typical lattice values for Fe_3_O_4_ and γ-Fe_2_O_3_.

To investigate structure–magnetization relationships, we calculated lattice parameters for each as-synthesized and phase-transferred sample and plotted them against their magnetization values (Fig. 7b). Fe(acac)_3_ systems cluster at *a* = 8.38–8.40 Å, close to Fe_3_O_4_, and retain high *M*_s_ ∼ 110 A m^2^ kg^−1^ even after phase transfer. This indicates that base bath, oxidative cleavage, and ligand exchange (NaCit or TMAH) do not significantly alter the spinel lattice or phase composition. In contrast, Fe(CO)_5_-derived IONPs cluster at *a* = 8.34–8.36 Å, closer to γ-Fe_2_O_3_, regardless of phase-transfer route, and consistently display lower magnetization. FeOl-based IONPs span *a* = 8.34–8.40 Å, implying that phase fraction (Fe_3_O_4_*vs.* γ-Fe_2_O_3_) depends on ligand/synthesis conditions; FeOl_NaOl tends to lie nearer the Fe_3_O_4_ band with slightly higher *M*_s_ than FeOl_OA.

Post-transfer, FTIR was used to understand the surface chemistry of Fe(acac)_3__OA/OAm/HDD and FeOl_OA IONPs (SI Fig. S9a and b). For Fe(acac)_3__OA/OAm/HDD, the Fe–O band shifts from ∼545 to ∼566 cm^−1^ after all phase transfers suggesting a change in the surface iron coordination environment upon ligand removal/exchange.^44^ In FeOl_OA, a broadened Fe–O band centered near ∼605 cm^−1^ red-shifts and sharpens to ∼583 cm^−1^, reflecting altered surface coordination. The distinct peak at 1702 cm^−1^ (C=O stretch of free oleic acid), prominent in FeOl_OA, is absent or less pronounced in Fe(acac)_3__OA/OAm/HDD, indicating physically adsorbed oleic acid bilayers on FeOl_OA.^45^ Elevated organic content in MP-AES (SI Sec. A.3.2 and Fig. S4) supports this bilayer arrangement. Changes in C–H symmetric/asymmetric vibrations (2850–2980 cm^−1^) further validate the success of phase transfer treatment. For Fe(acac)_3__OA/OAm/HDD, these peaks are substantially reduced after TMAH, base bath, and oxidative cleavage, indicating removal/replacement of alkyl chains. For FeOl_OA, oxidative cleavage and NaCit exchange markedly reduce both C–H and C=O bands, confirming efficient oleic-acid removal. During oxidative phase transfer, a reduction in C–H intensity is consistent with double-bond cleavage and probably leads to the formation azelaic/nonanoic acids which then make the particles hydrophilic.^9^ Hydrophobic IONPs showed carboxylate bands near 1512 and 1412 cm^−1^, shifting to ∼1656 and ∼1567 cm^−1^ post-transfer, indicating altered carboxylate coordination. In NaCit exchange, broad COO^−^ bands at ∼1622 and ∼1399 cm^−1^ provide evidence of citrate binding over the surface of IONPs.

Overall, all four phase-transfer methods successfully converted hydrophobic IONPs into stable aqueous dispersions without altering particle morphology, crystallinity, or magnetization. No systematic differences were observed in *H*_d_ size, AC-derived size, or *ζ*-potential values across methods. All samples exhibited sizes below 200 nm and strongly negative *ζ*-potential values, especially in Milli-Q water, which confirms the good colloidal stability.

From a practical standpoint, oxidative cleavage and base-bath-assisted methods are preferred for scalability, as both allow full-batch processing. Oxidative cleavage achieves complete transfer within ∼2 hours but requires complex reaction control, whereas the base-bath method is operationally simpler but requires longer processing times (∼24 hours). In contrast, NaCit and TMAH-based ligand exchanges are currently limited to 5–50 mg batches, and scaling is complicated by batch-to-batch variability and dependence on sonication parameters (type, power, vessel geometry).^46^ TMAH-treated nanoparticles also showed strong adhesion to plastic lab ware, causing material loss and necessitating the exclusive use of glassware.

### 3.4 Application: MRI performance of selected IONPs

One important biomedical application for IONPs is as MRI contrast agent. IONPs can act as both positive (*T*_1_) and negative (*T*_2_) MRI contrast agents. The quantitative measures for how efficiently MRI contrast agent shorten *T*_1_ and *T*_2_ proton relaxation times are coined the *T*_1_ and *T*_2_ relaxivity, referred to as *r*_1_ and *r*_2_, respectively. We measured *r*_1_ and *r*_2_ for two selected phase-transferred IONPs in a 7 T scanner. The choice of IONPs was made based on large differences between their magnetic saturation values. We found that both these IONPs exhibited high *T*_2_ relaxivities at this field strength, especially FeOl_NaOl. The *T*_1_ relaxivities were very low for both agents, resulting in very high *r*_2_/*r*_1_ ratios, see Fig. 8. For MRI contrast agents to be *T*_1_ or positive contrast agents, their *r*_2_/*r*_1_ should be low and close to the theoretical ideal of 1, while agents with *r*_2_/*r*_1_ larger than approximately 10–20 are typical *T*_2_ contrast agents.^47^ Increasing IONP size and aggregation significantly increases *r*_2_.^48^ Hence, very small IONPs (typically with magnetic cores below 5 nm) have a relatively low *r*_2_/*r*_1_ ratio and can act as rather powerful *T*_1_ contrast agents,.^49^ In contrast, larger IONPs aggregates have a high *r*_2_/*r*_1_ ratio and act as *T*_2_ contrast agents. In the current study, the IONPs increased in size from approximately 10 nm to around 40 nm upon IONP phase transfer, a result of IONP aggregation during the phase transfer process. Hence, this relatively large size explains the high *r*_2_/*r*_1_ ratios we measured. Combined with the high *r*_2_ of these phase-transferred IONPs, this demonstrates that these agents are powerful *T*_2_ contrast agents at 7 T. Clinically approved iron oxides like Feridex, Resovist and AMI-227, have *r*_1_ in the range of 10–25 and *r*_2_ in the range of 50–150 mM^−1^ s^−1^ at clinical field strengths.^50^*T*_1_ relaxivities (*r*_1_) of iron oxides typically decrease multiple fold from 1.5 to 7 Tesla, while *T*_2_ relaxivities typically display a plateau or an increase from 1.5 to 7 T.^51–53^ Considering this, it can be anticipated that the *r*_2_ for our iron oxides is in a similar range as for clinically approved iron oxides. As we measured very low *r*_1_ in the current study, our iron oxides agents will presumably be potent *T*_2_ contrast agents also at clinical field strengths. Although for quantitative MRI, *T*_1_ contrast agents are needed, for applications requiring high sensitivity, such as cell tracking^54^ or metastasis detection,^55,56^ powerful *T*_2_ contrast agents are critically important.^57^ Hence, our IONPs may find a role in such applications. Further optimization and understanding of the phase transfer approaches may allow for better control of the aggregation behavior upon phase transfer and can potentially broaden the biomedical utility of our agents.

**Fig. 8:**
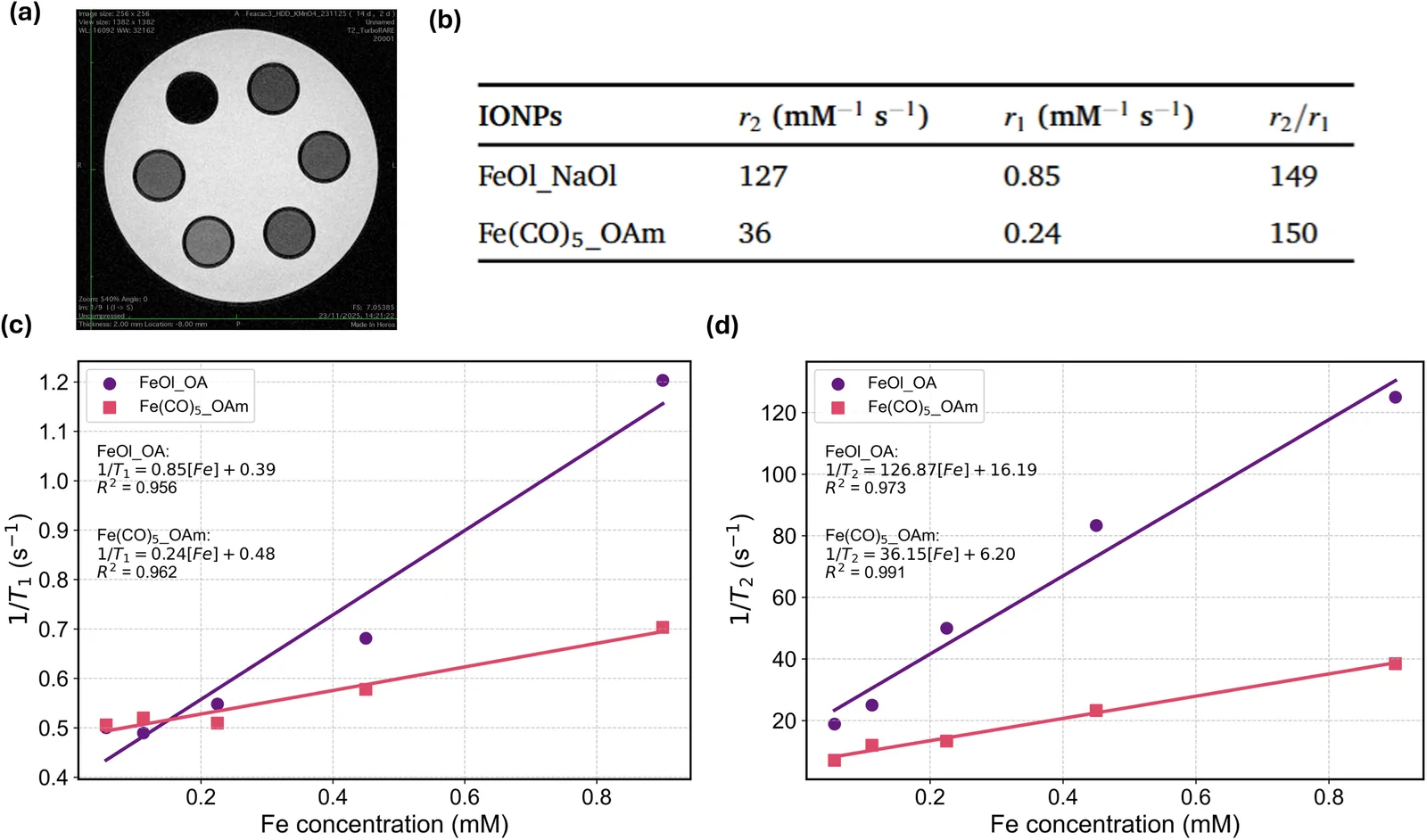
Relaxivity measurements of selected IONPs. (a) A cross-section MR image of the phantom holder with small NMR tubes containing the IONPs fixed inside a larger tube filled with water. (b) Relaxivities and *r*_2_/*r*_1_ ratios for the selected IONPs. (c and d) 1/*T*_1_ and 1/*T*_2_ plotted as a function of Fe concentration. The slope of these plots represents the relaxivities with units mM^−1^ s^−1^.

## 4 Conclusion

This study provides a comprehensive evaluation of IONP synthesis by thermal decomposition using three different precursor types and subsequent phase-transfer by four different strategies. The results highlight the critical role of precursor chemistry in dictating lattice parameters, phase composition, crystallinity, and magnetic performance. Among the investigated systems, Fe(acac)_3__OA/OAm/HDD yielded Fe_3_O_4_ NPs with superior crystallinity and magnetization (*M*_s_ ∼ 112 A m^2^ kg^−1^ at 300 K). FeOl afforded tunable size/shape but mixed phases and sub-bulk *M*_s_, while Fe(CO)_5_-derived NPs suffered magnetic degradation due to amorphous shells.

Across phase-transfer routes, oxidative cleavage and base bath emerged as preferred for processing larger batches while preserving magneto-structural properties. Ligand exchanges (NaCit, TMAH) are effective for small batches but are sensitive to sonication and scale-up parameters. Selected phase transferred IONPs produced by oxidative cleavage method also showed promise as *T*_2_ contrast agents.

This systematic exploration underscores the interplay among synthesis parameters, precursor selection, and phase-transfer chemistry in tailoring IONPs for application-specific performance. The framework provided here should facilitate the rational design of hydrophilic IONPs with optimized magnetic and colloidal properties for nanomedicine and beyond.

## Author contributions

Sulalit Bandyopadhyay funding acquisition, project administration, supervision, writing – review & editing, and formal analysis. Haroon Zafar visualization, writing – review & editing, writing – original draft, data curation, investigation, and formal analysis. Vyshnav PS writing – review & editing, writing – original draft, data curation, investigation, and formal analysis. Tina Bergh writing – review & editing, investigation, and formal analysis. Davide Peddis, Sawssen Slimani writing – review & editing, and investigation. Sjoerd Hak writing – review & editing, and formal analysis.

## Conflicts of interest

Sulalit Bandyopadhyay is an employee and owns stock in Lybe Scientific AS.

## Supplementary Material

NA-008-D6NA00313C-s001

## Data Availability

The data supporting this article have been included as part of the supplementary information (SI). Supplementary information is available. See DOI: https://doi.org/10.1039/d6na00313c.
