# Supplementary material for: Phase transfer studies of iron oxide nanoparticles: characterization based insights for applications
Source: Nanoscale Adv. 2026 Aug 19;8(20):5908–24. doi: 10.1039/d6na00313c (PMC13549772; doi:10.1039/d6na00313c)
Supplement: Supplementary file 1 [file NA-008-D6NA00313C-s001.pdf]

This journal is © The Royal Society of Chemistry 2026

A Supporting Information

A.1 XRD and Calculation of Lattice Parameters and Crystallite Size

We characterized the XRD patterns of all the as-synthesized and phase-transferred IONPs, as shown in Figure S1.

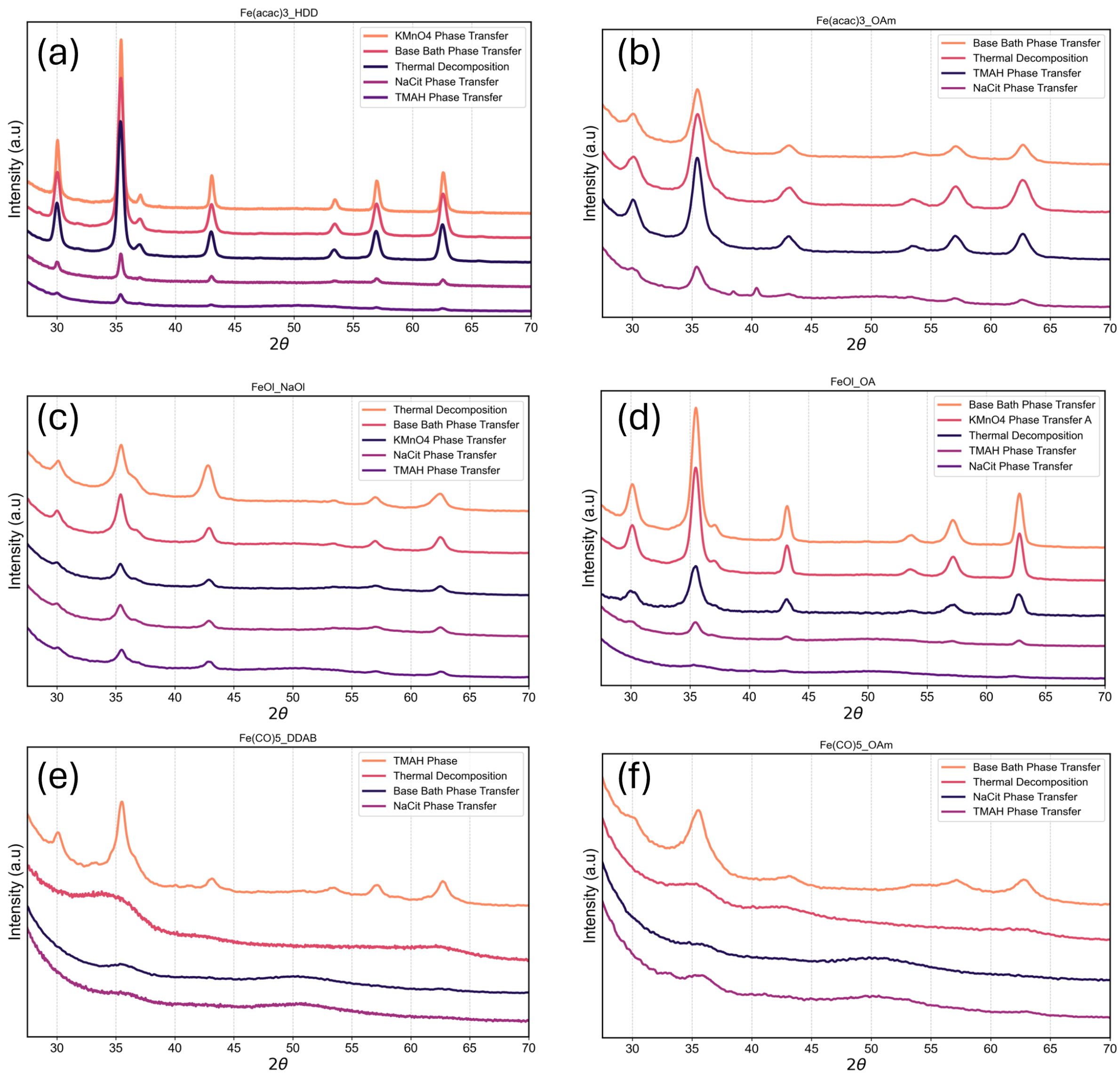

Fig. S1 XRD patterns of IONPs synthesized using different precursors and subjected to various phase transfer methods. (a) Fe(acac)$_3$_OA/OAm/HDD, (b) Fe(acac)$_3$_OAm, (c) FeOl_NaOl, (d) FeOl_OA, (e) Fe(CO)$_5$_DDAB, and (f) Fe(CO)$_5$_OAm nanoparticles. Each panel compares thermal decomposition with oxidative cleavage, base bath, NaCit, and TMAH phase transfer methods.

Lattice parameters and crystallite sizes were calculated using the Pawley fitting method implemented in Topas software. This structure-less, whole-pattern fitting technique models XRD data by refining the positions and intensities of diffraction peaks without requiring detailed information about the structural model. The diffraction patterns were indexed to the cubic crystal structure of IONPs, corresponding to the Fd$\bar{3}$m (space group 227) symmetry. The peak profiles were modeled using a pseudo-Voigt (PV) function, which accounts for both Gaussian and Lorentzian contributions to peak broadening. Representative examples of XRD patterns for as-synthesized and phase-transferred IONPs fitted using the Pawley method are provided in Figure S2.

The quality of the refinement was evaluated using standard indicators, including the goodness-of-fit (GOF) (χ^2), the weighted profile R-factor (R_{wp}), and the expected R-factor (R_{exp}). A χ^2 value close to 1 suggests a good fit between the experimental and calculated data.

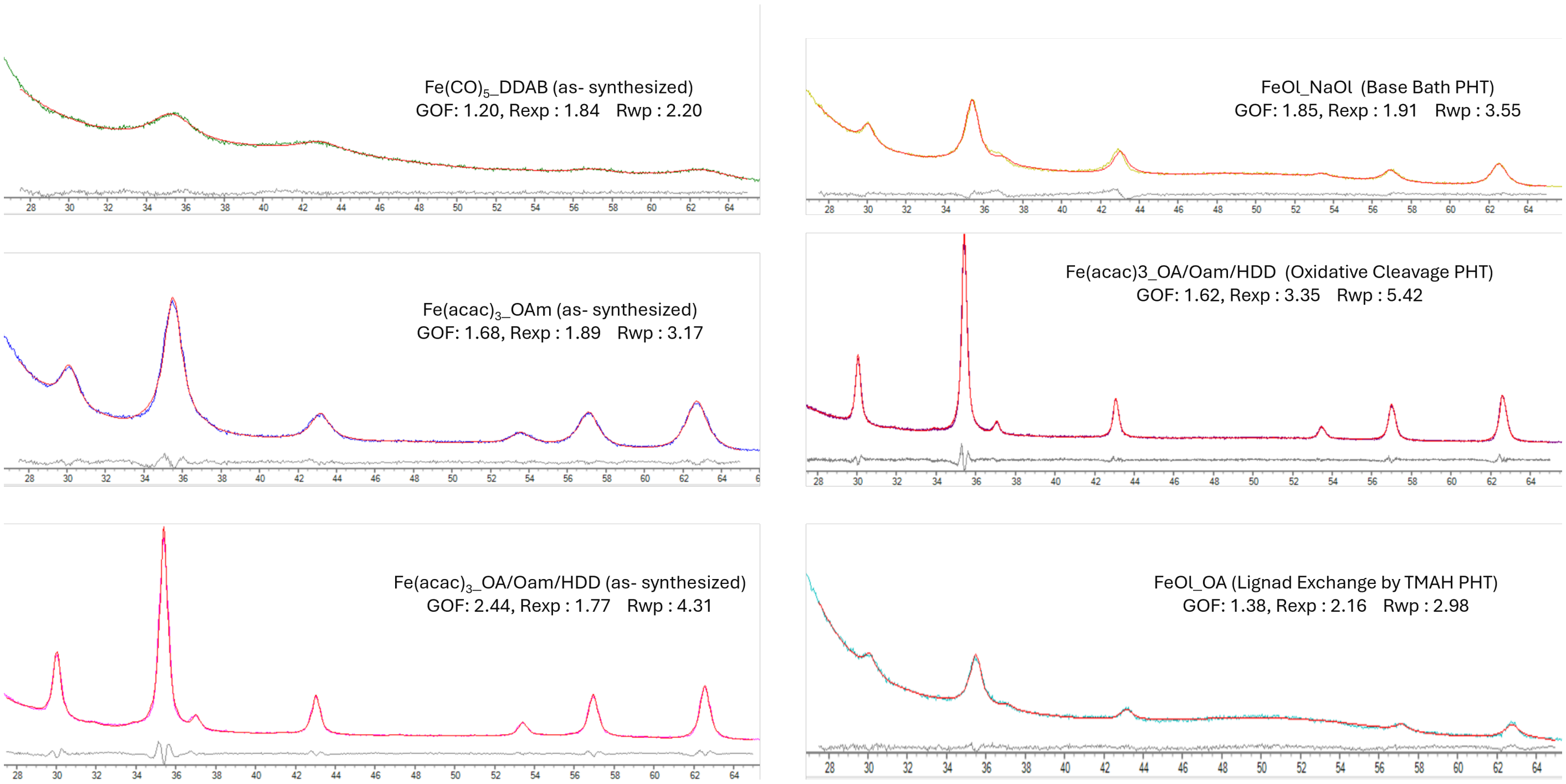

Fig. S2 Representative examples of XRD pattern for as-synthesized and phase-transferred IONPs fitted using the Pawley method.

Precursor	Name	Lattice Constant (Å)
Fe(acac)$_3$_OA/OAm/HDD	Thermal Decomposition	8.390
	$KMnO_4$ Based Oxidative Cleavage	8.382
	NaOH Assisted Base Bath	8.385
	Sodium Citrated Based Ligand Exchange	8.385
	TMAH Based Ligand Exchange	8.396
Fe(acac)$_3$_OAm	Thermal Decomposition	8.380
	$KMnO_4$ Based Oxidative Cleavage	8.383
	NaOH Assisted Base Bath	8.367
	Sodium Citrated Based Ligand Exchange	8.380
	TMAH Based Ligand Exchange	8.373
FeOl_NaOl	Thermal Decomposition	8.374
	$KMnO_4$ Based Oxidative Cleavage	8.397
	NaOH Assisted Base Bath	8.394
	Sodium Citrated Based Ligand Exchange	8.394
	TMAH Based Ligand Exchange	8.386
FeOl_OA	Thermal Decomposition	8.353
	$KMnO_4$ Based Oxidative Cleavage	8.355
	NaOH Assisted Base Bath	8.357
	Sodium Citrated Based Ligand Exchange	8.345
	TMAH Based Ligand Exchange	8.365
Fe(CO)$_5$_DDDAB	Thermal Decomposition	8.347
	NaOH Assisted Base Bath	8.351
	Sodium Citrated Based Ligand Exchange	8.343
	TMAH Based Ligand Exchange	8.375
Fe(CO)$_5$_OAm	Thermal Decomposition	8.344
	NaOH Assisted Base Bath	8.345
	Sodium Citrated Based Ligand Exchange	-
	TMAH Based Ligand Exchange	-
	$KMnO_4$ Based Oxidative Cleavage	-

Table S1 Lattice constant comparison for various precursors and treatments.

A.2 Transmission Electron Microscopy

The SAED patterns were processed using the open-source python library pyxem[21], which builds on hyperspy[20]. The reciprocal scale was calibrated using a standard polycrystalline Au specimen. For each SAED pattern, the central cross (coming from the gap between the four chips constituting the quad detector) was corrected for, and the direct beam was centered. Polar re-projection was performed, and a 1D integrated intensity profile was extracted from the region indicated by black dashed lines in Figure 4. The Bragg peaks were indexed with respect to the cubic crystal structure of magnetite ($Fd\bar{3}m$, $a = 8.39$ Å).

A.3 Characterization of Magnetic Properties of IONPs

The saturation magnetization of as-synthesized and phase-transferred IONPs was measured using a VSM with dried IONPs loaded into gel capsules. Given the variability in dried masses across samples, normalization was performed using three methods: dried mass, mass fraction determined by TGA, and iron content quantified via MP-AES.

Mass-based normalization alone can be misleading, as samples with higher iron oxide content exhibit higher magnetization, while those with substantial organic material show reduced values. As shown in Table S2, significant variability in magnetization was observed before correcting for iron content. After normalization, saturation magnetization values were consistent across sample types.

**Sample Name**	**Ms (dry mass) (Am^2/Kg)**	**Ms (of Fe) (Am^2/Kg)**
FeOl_OA	11<br>21	77<br>79
$Fe(acac)_3$_OA/OAm/HDD	76<br>55	117<br>116

Table S2 Comparison of saturation magnetization measured at 300K and normalized for dry mass and Fe for different samples.

A.3.1 Field Dependence of Magnetization at 5K

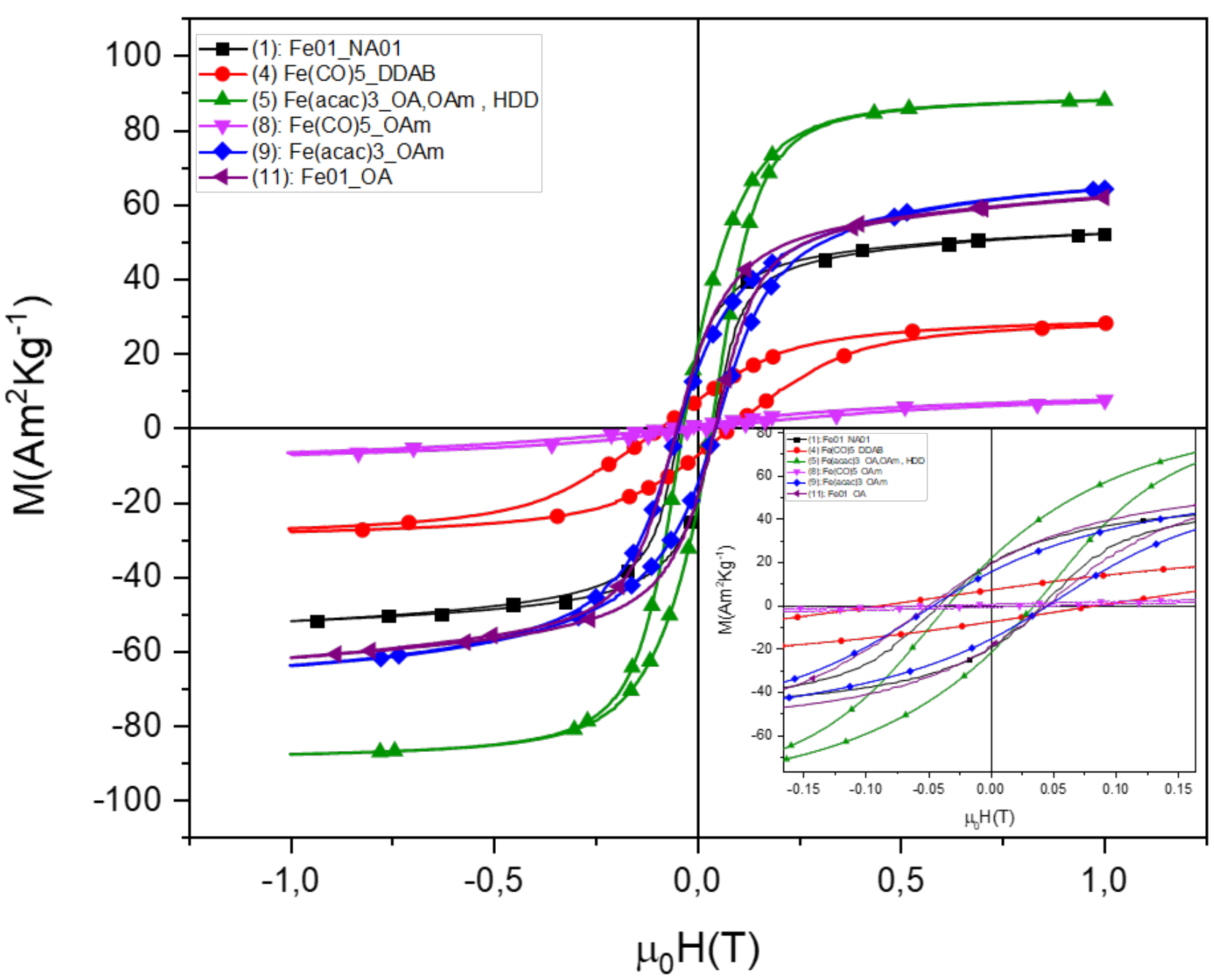

Fig. S3 Field dependence of magnetization at 5K; inset detail at low field.

A.3.2 Qualitative vs Quantitative Measurement of Organic Content

MP-AES provides quantitative measurements of the organic content in IONP samples, while FTIR offers complementary insights into surface chemistry and the thickness of coating by examining the intensity ratio of C–H bands (2800–2950 cm^{-1}, CH_2 and CH_3 stretching) to Fe–O bands. As shown in Figure S4, FeOl-derived nanoparticles, with the highest organic content (64%-92% by MP-AES), exhibited

Sample	Ms $(Am^2/Kg)_{5K}$	Hc (T)	Mr/Ms
Fe0l_NaOl	59(5)	0.041	0.2
Fe0l_OA	75(5)	0.046	0.25
$Fe(CO)_5$_OAm	—-	0.046	—-
$Fe(CO)_5$_DDAB	32(4)	0.086	0.22
$Fe(acac)_3$_OAm	77(2)	0.045	0.19
$Fe(acac)_3$_OA,OAm,HDD	89(7)	0.034	0.24

Table S3 Saturation magnetization (Ms), reduced remanent magnetization (Mr/Ms), coercivity (μ0Hc), at 5 K. Uncertainties are given in parentheses.

the most pronounced C–H to Fe–O intensity difference. In contrast, $Fe(acac)_3$-derived nanoparticles displayed a strong Fe–O peak at 545 cm^{-1}, with a less prominent C–H to Fe–O intensity difference, reflecting low organic content (8-17%) and high crystallinity. $Fe(CO)_5$-derived nanoparticles showed intermediate C–H to Fe–O ratios, corresponding to an organic content of 21%–40% and a blend of crystalline and amorphous features. This correlation between C–H and Fe–O peak intensities provides a simple qualitative method to estimate the organic and inorganic composition of nanoparticles.

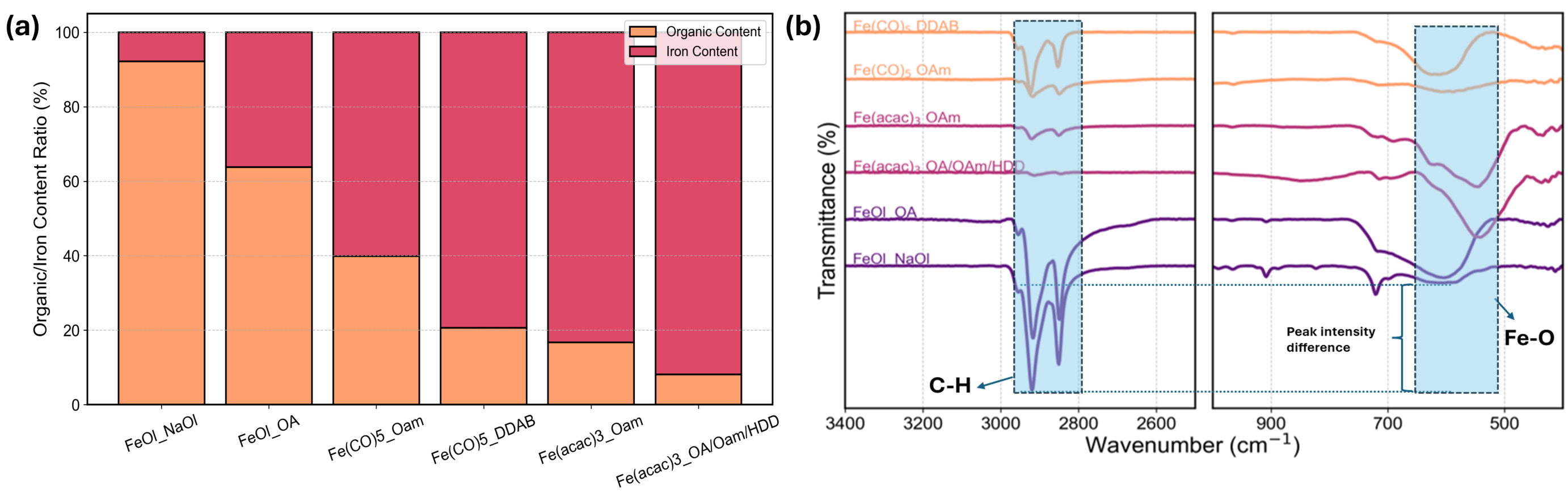

Fig. S4 (a) Comparison of organic and iron content ratios in all IONPs synthesized, calculated using MP-AES analysis. (b) FTIR spectra of all IONPs synthesized.

A.3.3 TGA Analysis

The TG curves for most samples exhibited a single weight-loss step, indicative of a straightforward decomposition process (Fig. S5). In contrast, the $Fe(CO)_5$_DDAB sample displayed two distinct weight-loss steps, suggesting a more complex thermal degradation mechanism. The residual masses derived from the TGA data varied significantly across samples (Tab. S4), reflecting differences in the organic phase content.

A.4 Surfactant role in $Fe(acac)_3$_OAm_OA_HDD sysnthesis

To clarify the roles of OA, OAm, and HDD, we performed control experiments using each component individually (with $Fe(acac)_3$ in benzyl ether). OA alone yielded cubic particles, while OAm alone produced pseudo-spherical particles, consistent with selective facet binding by OA (carboxylate) vs. relatively isotropic binding by OAm (amine).[16] Reported binding energies at an OA:OAm ratio of 4:1 (as used here) are ~ -6500 kcal mol^{-1} versus ~ -3000 kcal mol^{-1} for OA alone,[57] suggesting a co-adsorption of both ligands. Overall, these observations indicate that the coexistence of cubic and truncated morphologies arises from the complex interplay between ligand binding competition, solvent decomposition, and reduction kinetics, rather than from a single dominant shape-directing mechanism.

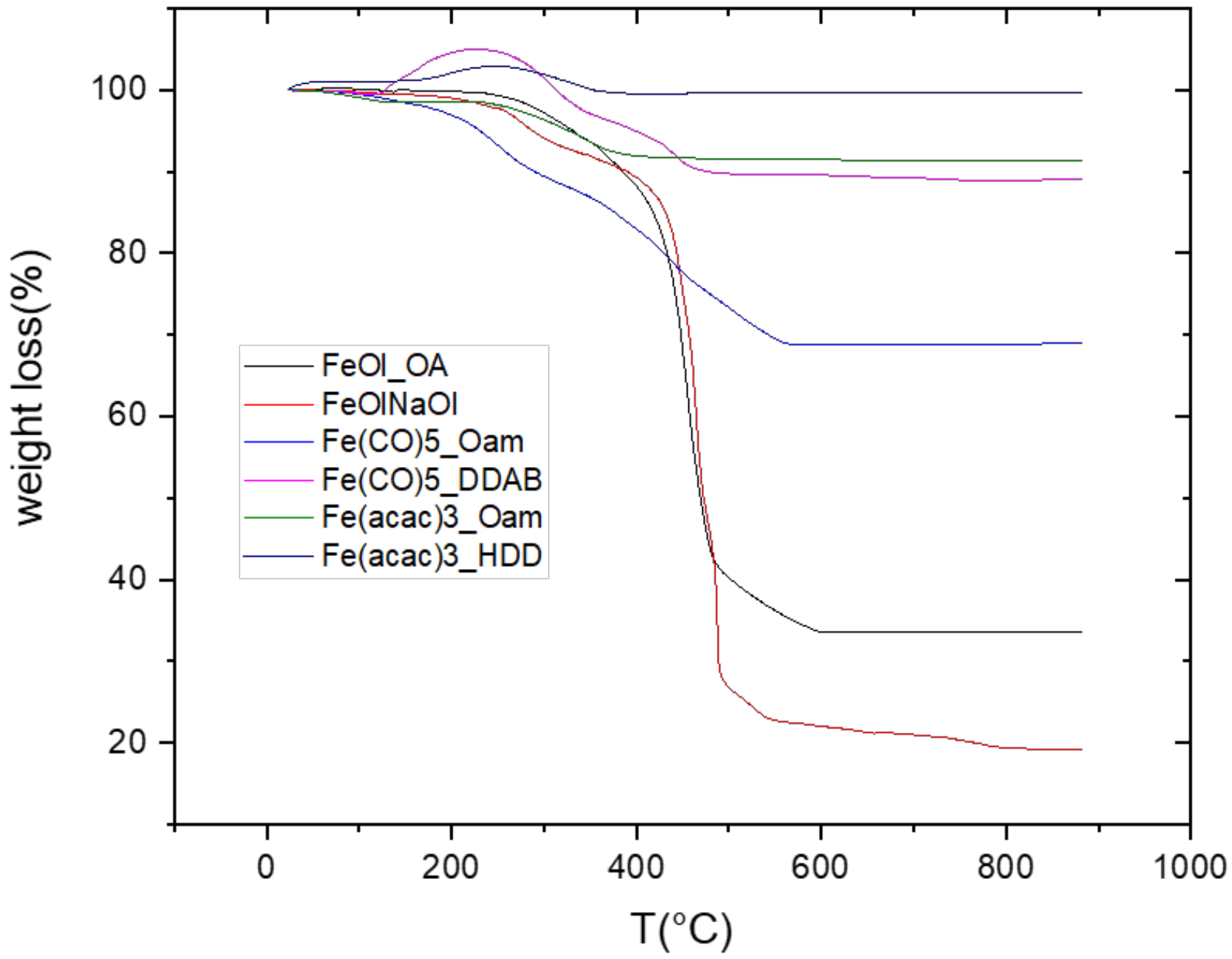

Fig. S5 The TGA thermogram curves of organic ligand-coated magnetic nanoparticles.

Sample	TG (%) in Organic Phase
FeOI_NaOl	82.0
FeOl_OA	67.5
$Fe(CO)_5$_OAm	32.0
$Fe(CO)_5$_DDAB	11.0
$Fe(acac)_3$_OAm	9.0
$Fe(acac)_3$_OA/OAm/HDD	2.0

Table S4 TG analysis showing the percentage of organic phase content for different samples.

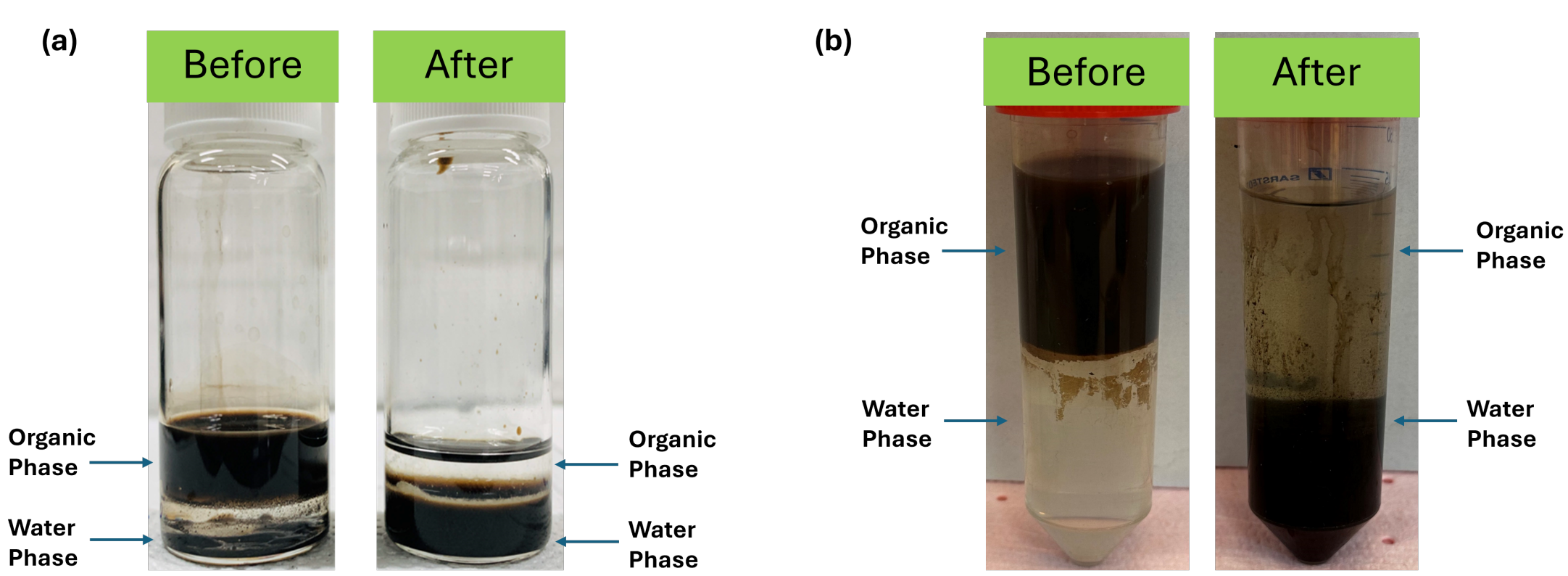

Fig. S6 (a) Photographs of FeOl_OA before and after phase transfer using Na Citrate ligand exchange. (b) $Fe(acac)_3$_OA/OAm/HDD before and after phase transfer using base bath assisted method.

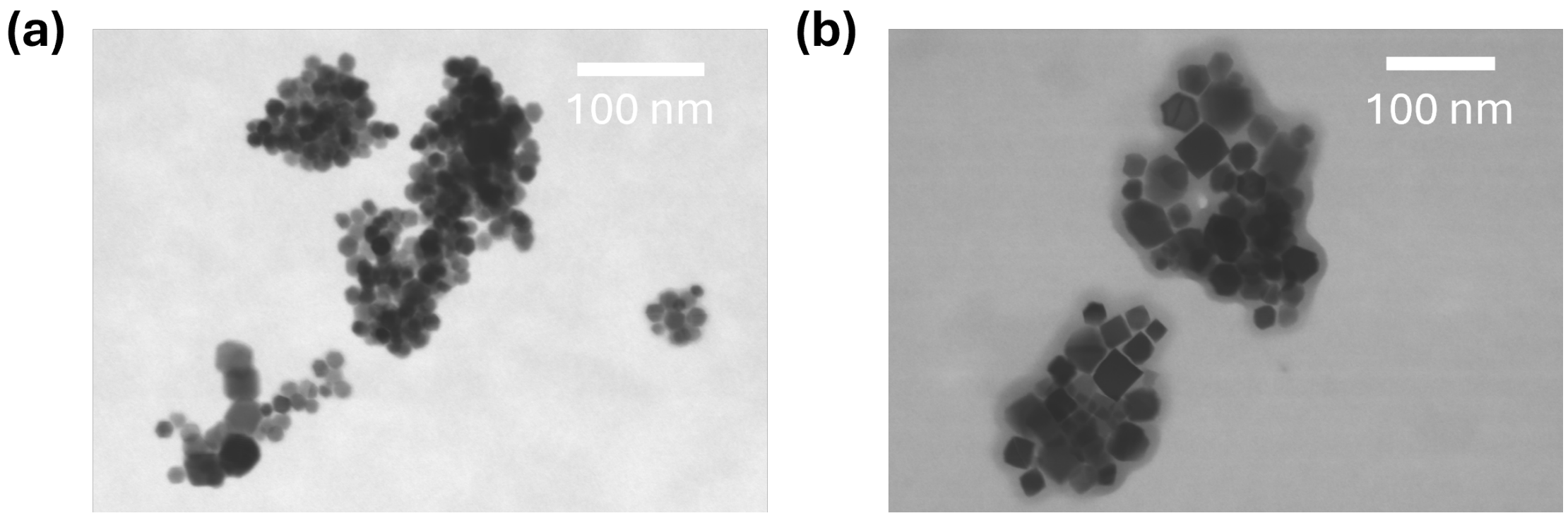

Fig. S7 (a) STEM images of $Fe(acac)_3$_OA/OAm/HDD IONPs phase transferred by base-bath assisted method and (b) lignad exchange using NaCit method.

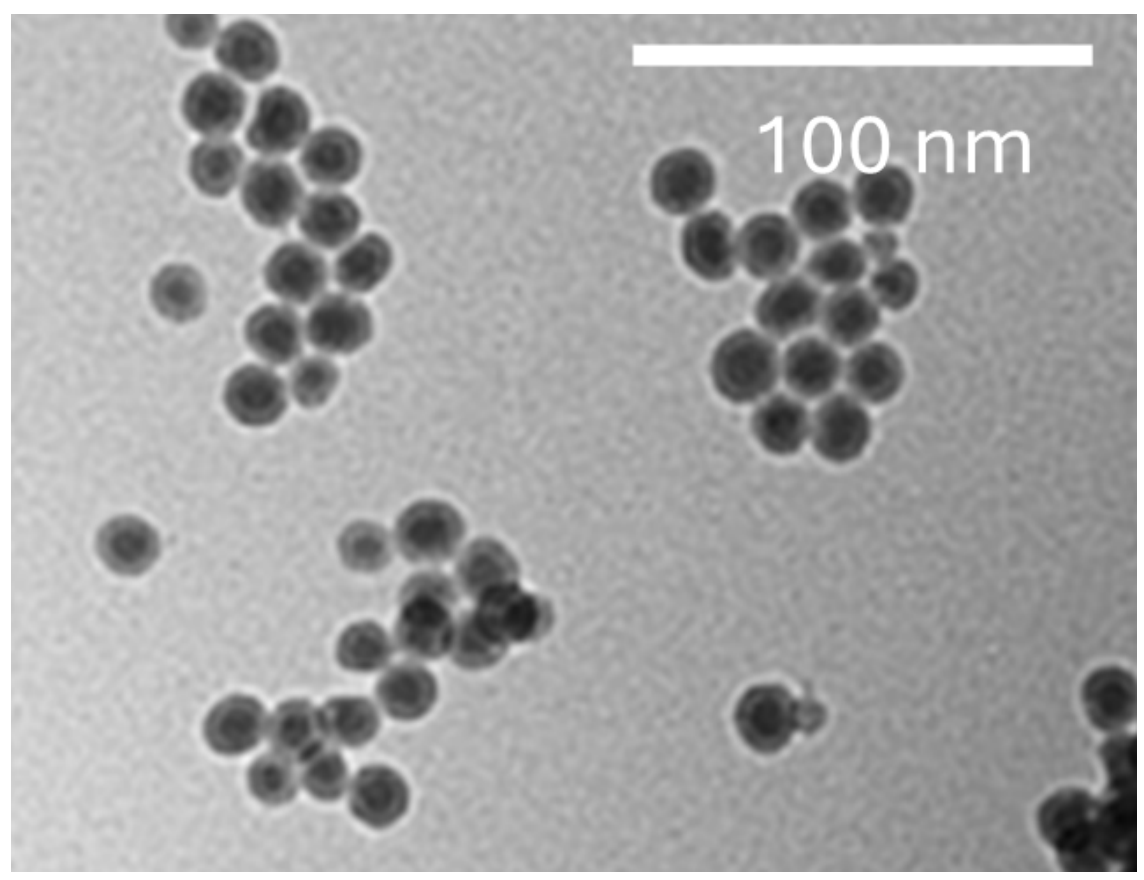

Fig. S8 BF-TEM image of $Fe(CO)_5$_OAm IONPs showing a core-shell morphology.

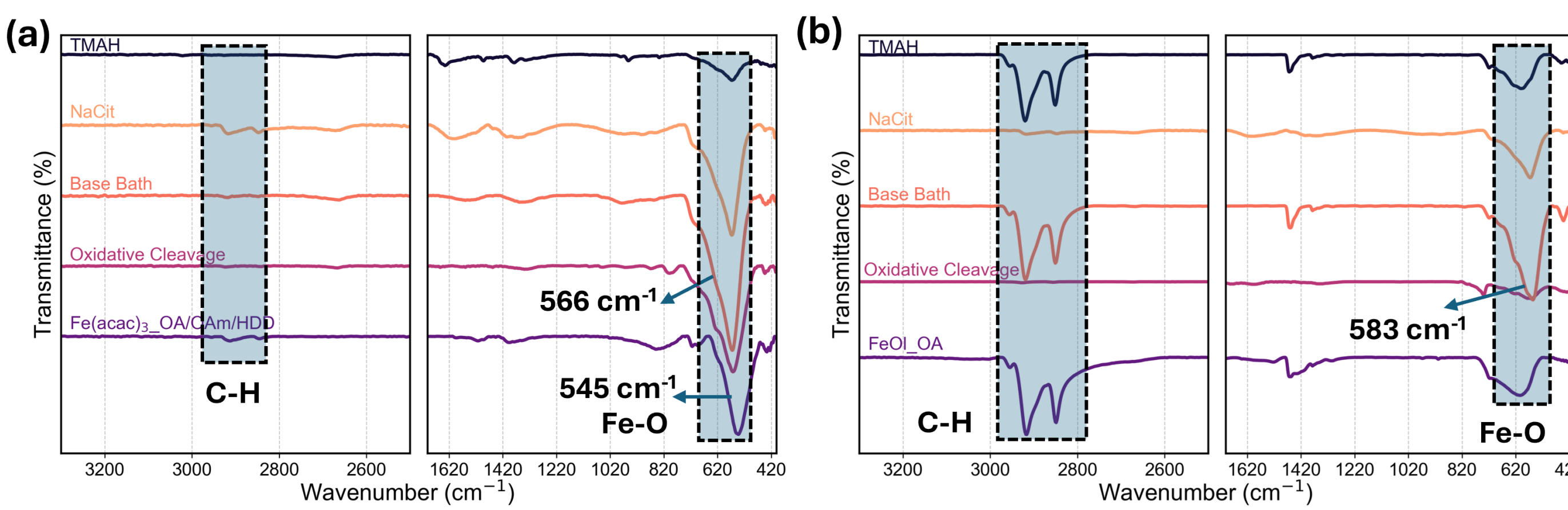

Fig. S9 FTIR spectra of $Fe(acac)_3$_OA/OAm/HDD (a) and FeOl_OA (b) before and after phase transfer (TMAH, NaCit, base bath, oxidative cleavage.

A.5 Phase-Transferred IONPs

A.6 Fe-based concentrations used for and after phase transfer

For all phase-transfer experiments, a fixed IONP input mass was used: 50 mg for oxidative cleavage, base bath, and TMAH, and 5 mg for NaCit. After transfer and washing, the particles were redispersed in 10 mL Milli-Q water (50 mg routes) or 2 mL Milli-Q water (NaCit), corresponding to nominal loadings of 5 mg IONP mL^{-1} and 2.5 mg IONP mL^{-1}, respectively. Because the organic/inorganic content differed between samples, equal IONP masses did not correspond to equal Fe masses; the input Fe mass and the resulting

nominal post-transfer Fe concentrations for each sample, derived from the MP-AES Fe contents, are given in Table S5. Values assume full recovery; actual concentrations may be slightly lower due to losses during magnetic separation and washing.

Sample	Fe content (%)	Fe in 50 mg IONPs (mg)	Conc., 50 mg route (mg Fe mL^{-1})	Fe in 5 mg IONPs (mg)	Conc., NaCit route (mg Fe mL^{-1})
FeOl_OA	26.2	13.10	1.31	1.31	0.66
FeOl_NaOl	5.7	2.85	0.29	0.29	0.14
$Fe(CO)_5$_OAm	43.5	21.75	2.18	2.18	1.09
$Fe(CO)_5$_DDAB	57.5	28.75	2.88	2.88	1.44
$Fe(acac)_3$_OAm	60.3	30.15	3.02	3.02	1.51
$Fe(acac)_3$_OA/OAm/HDD	66.5	33.25	3.33	3.33	1.66

Table S5 Nominal Fe concentrations of IONP dispersions after phase transfer. Fe concentrations were calculated from the MP-AES-derived Fe contents, the IONP input mass, and the final redispersion volume. Oxidative cleavage, base-bath-assisted phase transfer, and TMAH used 50 mg IONPs redispersed in 10 mL Milli-Q water. NaCit used 5 mg IONPs redispersed in 2 mL Milli-Q water. Values assume complete recovery after phase transfer and washing; actual concentrations may be lower due to material losses during magnetic separation and washing. The listed concentrations apply only to the phase-transfer routes performed for each sample.
